# Decoding P300 as a shared neural mechanism for oddball target detection and working memory updating

**DOI:** 10.1016/j.isci.2026.114757

**Published:** 2026-01-21

**Authors:** Weixing Yang, Shuoqi Xiang, Bijuan Huang

**Affiliations:** 1 Faculty of Psychology, Shandong Normal University, Jinan, Shandong, China; 2 Shandong Provincial Key Laboratory of Brain Science and Mental Health, Jinan, Shandong, China; 3 School of Mental Health and Psychological Sciences, Anhui Medical University, Hefei, Anhui, China; 4 School of Education and Psychology, University of Jinan, No. 336, West Nanxinzhuang Road, Shizhong District, Jinan City, Shandong, China

**Keywords:** Systems neuroscience, Cognitive neuroscience, Biocomputational method

## Abstract

The P300 is a well-established neurophysiological component that has been extensively investigated within the oddball paradigm and is commonly interpreted as reflecting context or working memory (WM) updating. This interpretation implies that the P300 elicited by oddball target detection shares neural mechanisms with the P300 elicited by WM. To test this hypothesis, we applied multivariate pattern analysis to perform cross-task decoding between EEG patterns during an oddball task and an n-back task, a classical WM paradigm. We found that classifiers trained on EEG patterns from the oddball task successfully decoded those from the n-back task, and vice versa, with the effect concentrated in the parietal/occipital region within the P300 time window. These findings suggest that the P300 serves as a shared neural mechanism for oddball target detection and WM updating, supporting the WM updating account of the P300 from the perspective of neural representation.

## Highlights

• EEG MVPA enables cross-task decoding between oddball and n-back tasks
• P300 serves as a shared neural mechanism for oddball target detection and WM updating
• Shared P300 effect remains robust after controlling for WM load
• Cross-task MVPA supports the context updating theory of the P300

## Graphical abstract

**Figure:**
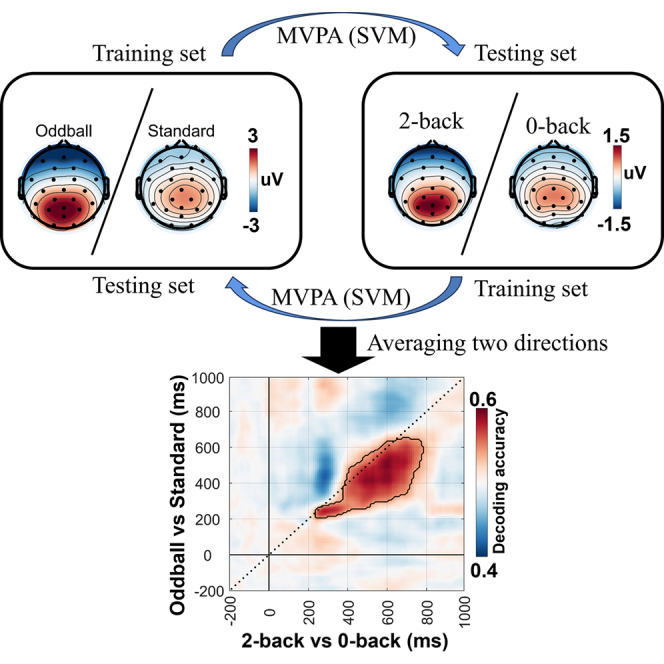

## Introduction

The P300 is one of the most extensively studied components of the event-related potential (ERP). It is characterized by a positive deflection occurring approximately 300–600 ms after stimulus onset, predominantly distributed over parietal regions. The P300 comprises two sub-components, P3a and P3b, with P3b often referred to as the P300.^1^ Generally, research on the P300 employs signal detection paradigms, among which the “oddball” task is the most widely used.^2^ In this paradigm, rare and task-relevant events (i.e., oddball stimuli) are embedded within a stream of frequent and task-irrelevant events (i.e., standard stimuli). Consistent findings have shown that oddballs elicit a larger P300 amplitude than standards.^3^^,^^4^^,^^5^^,^^6^

The most influential explanation for the P300 is the context updating theory.^7^ This theory proposes that the P300 is elicited when the brain detects a change or mismatch between a task-relevant stimulus and its immediate context (i.e., preceding standard stimuli), prompting the updating of the “schema” or mental model of the environment stored in memory.^7^ Although Donchin did not explicitly use the term “working memory” (WM), subsequent studies have widely interpreted the P300 as reflecting WM updating.^1^^,^^8^^,^^9^ This suggests a possibility: if the P300 reflects WM updating, then the neural basis of the P300 elicited by the detection of oddball targets and by WM should substantially overlap. In other words, the P300 may be a shared neural mechanism between the detection of oddball targets and WM updating. Investigating this question not only helps to deepen our understanding of the cognitive functions of the P300 but also provides theoretical guidance for the practical applications of P300, such as developmental and aging studies^10^^,^^11^^,^^12^^,^^13^^,^^14^^,^^15^ and psychosis risk syndrome.^16^^,^^17^^,^^18^

WM refers to a limited-capacity memory system responsible for the temporary storage and manipulation of information.^19^ The n-back task, originally introduced by Kirchner,^20^ has become one of the most widely used experimental paradigms in cognitive neuroscience research on WM. Importantly, the n-back is presumably the most widely used WM updating task.^10^^,^^11^^,^^21^ As a continuous recognition task, the n-back requires participants to determine whether the current stimulus matches the one presented n trials earlier and to respond accordingly. Typically, n is set to 0, 1, 2, or 3 to manipulate the level of memory load.^11^^,^^22^^,^^23^^,^^24^^,^^25^ Notably, the P300 has been repeatedly observed in n-back tasks and is commonly interpreted as an index of WM updating.^1^^,^^12^^,^^26^^,^^27^^,^^28^^,^^29^^,^^30^ However, whether the P300 elicited by the detection of oddball targets (henceforth: oddball-P300) and that elicited by the n-back task (henceforth: n-back-P300) share neural mechanisms has not yet been systematically examined.

Several lines of indirect evidence support the hypothesis of shared neural mechanisms underlying the detection of oddball targets and WM updating. For example, Carrasco et al. examined the relationship between oddballs and WM performance.^31^ They found that individuals with larger P300 amplitude elicited by oddballs demonstrated more accurate memory performance. Additionally, the oddball-P300 and n-back-P300 show a high degree of similarity in scalp topography.^1^^,^^8^^,^^32^^,^^33^^,^^34^ In terms of neural generators, the oddball-P300 and n-back-P300 have been localized to similar sources, primarily within hippocampal structures.^24^^,^^35^^,^^36^ In developmental studies, the amplitude of the P300 decreases with age in the oddball task,^8^^,^^13^^,^^37^^,^^38^ and a similar trend has also been observed in WM tasks.^11^^,^^39^ Collectively, these findings suggest that the P300 may reflect WM updating.

However, some researchers have questioned the association between the P300 and WM updating. First, an analysis of task characteristics suggests that the classical oddball paradigm typically involves a series of stimuli in which 80% are the letter “X” and 20% are the letter “O.” The task requires responding to each letter with a corresponding button press.^40^ Responses are made immediately, so there is no need to maintain the identities of Xs or Os in WM. This suggests that the detection of oddball targets may not be directly related to WM. Second, from a theoretical standpoint, Verleger^41^ raised several critiques of the influential review by Polich.^1^ For example, Verleger pointed out that the brain regions generating the P300 differ from those typically associated with memory processing, and that there is no reliable difference in P300 amplitude between remembered and forgotten items.^41^ More recently, Verleger further challenged the plausibility of the context updating theory in accounting for simple oddball effects.^42^ He argues that “when proper updating is required, any adequate environmental model should certainly allow for the occurrence of both rare and frequent stimuli, as both types are inherently part of the model.” Therefore, it is unclear why encountering a rare stimulus would require more updating than a frequent one. This raises the question regarding the true functional role of the P300. Verleger argued that if a model fails to recognize the statistical structure of the environment and requires repeated updating whenever a rare event occurs, then it lacks theoretical utility.^42^ Moreover, in developmental research using the n-back task, Pelegrina et al. noted that the n-back-P300 does not align with the broader body of literature on the oddball-P300 studies, suggesting that the n-back-P300 might be more accurately described as a late positive potential (LPP).^11^ Third, empirical evidence also supports this line of skepticism. Specifically, one study reported smaller P300 amplitudes on alternation trials than on repetition trials, despite alternation imposing greater demands on WM updating.^43^ Subsequent research employed a reference-back task specifically designed to distinguish WM updating from other cognitive processes, and found no evidence that P300 is elicited when WM updating is required.^44^ Therefore, these findings suggest that the debate over whether the P300 reflects WM updating remains unresolved.

Two key reasons may underlie this controversy. First, although extensive literature has developed in both the oddball and WM domains, these two lines of work have largely evolved in parallel, with limited systematic theoretical or empirical integration. Consequently, the potential relationship or neural similarity between the P300 elicited in each domain has not been precisely quantified. Second, most prior studies have relied on univariate analyses, such as topography correlations or amplitude comparisons. Even when univariate analyses reveal overlapping scalp distributions across tasks, such spatial similarities do not necessarily reflect shared neural representations, as anatomically overlapping regions may contain functionally distinct neuronal populations.^45^^,^^46^^,^^47^ To address these issues, a promising approach is to assess whether the P300 elicited by the detection of oddball targets is functionally related to WM. Specifically, examining whether the oddball-P300 and the n-back-P300 share neural mechanisms may offer critical insights into this potential relationship.

To this end, from the perspective of neural representation, the present study employs multivariate pattern analysis (MVPA) to examine the shared neural mechanisms between the oddball and n-back tasks. MVPA refers to the application of machine learning classification algorithms to multiple electrodes (or voxels) data to decode the distributed neural representation patterns. It provides a sensitive method for quantifying the neural similarity across cognitive tasks.^48^^,^^49^^,^^50^^,^^51^^,^^52^^,^^53^ MVPA provides a principled and explicit measurement standard for quantifying shared neural representations.^4^^,^^54^ Importantly, as a powerful extension of MVPA, temporal generalization analysis (TGA) enables researchers to assess whether a particular neural representation pattern trained at a given time point (e.g., *t*_*1*_) can successfully classify patterns at the other time points (e.g., *t*_*2*_). Successful classification indicates that the neural representation pattern is sustained or reactivated over time, whereas failure indicates dynamic changes over time. This approach can be readily applied to cross-task decoding. Specifically, a classifier trained on the EEG patterns of conditions in one task (e.g., oddballs vs. standards) can be tested on the EEG patterns of conditions in another task (e.g., 2-back vs. 0-back). If the classification accuracy significantly exceeds chance level, it indicates a substantive similarity in the underlying neurocognitive events between the two tasks.^45^^,^^54^ The resulting temporal generalization matrix provides an intuitive time-resolved depiction of the extent to which neural mechanisms are shared between distinct tasks. This method has been applied to investigate the shared neural mechanisms across diverse cognitive domains. For example, Sassenhagen and Fiebach trained a classifier on the P300 EEG patterns elicited by the detection of oddball targets and successfully classified P600 EEG patterns elicited by syntactic violations, demonstrating that the P300 and P600 may share a substantial degree of neural mechanisms.^4^ Additionally, this method has revealed shared neural representations between visual perception and imagery^55^^,^^56^ and domain-general mechanisms across multiple tasks.^57^ Collectively, these studies suggest that cross-task decoding can reliably answer the core question of the present study.

**Figure 1:**
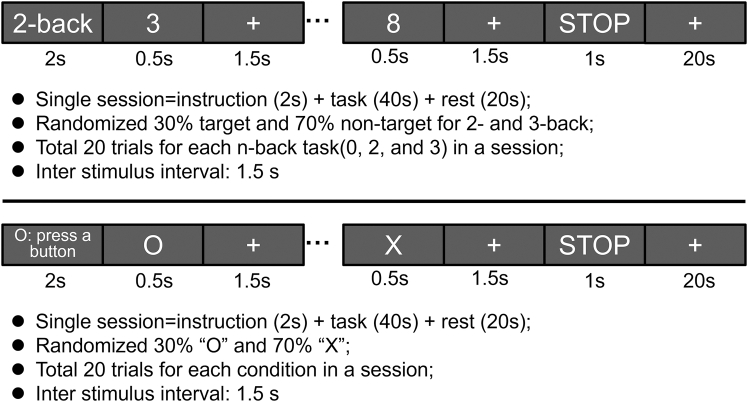
Timing sequence of the n-back and oddball experimental paradigms

In sum, this study aims to determine whether the oddball-P300 and n-back-P300 share neural mechanisms. The task procedures used in this study are illustrated in Figure 1. We expected that the cross-task decoding performance between oddball and n-back EEG data would be significantly higher than the chance level, especially within the P300 time window.

## Results

### Behavioral results

Since this study focused on the EEG effects and their relationships, the behavioral results were not assessed in detail. Additional behavioral data information can be found in Shin et al.^58^

### Event-related potential results

**Figure 2:**
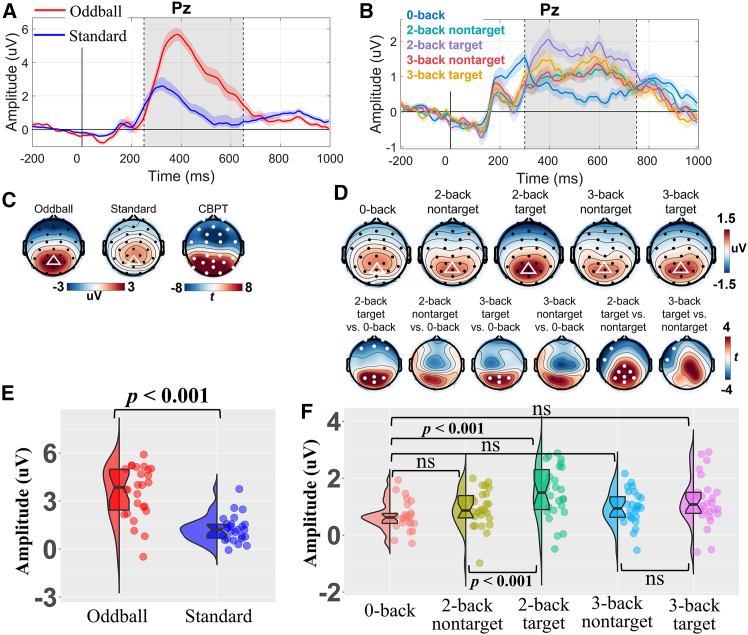
Grand-averaged ERP P300 waveforms of Pz and corresponding topographical maps (A and B) show the P300 waveforms for the oddball task (250–650 ms) and n-back task (300–750 ms), respectively. The time range between the vertical dashed lines in each waveform represents the P300 time window. The shaded areas represent the standard error of the mean (SEM). (C) The first two present the grand-average topographical maps of P300 mean amplitude for the oddball task. The Pz electrode is marked with a white triangle on the topographical maps. The third presents the *t*-value topographical map from the cluster-based permutation test, with significant electrodes marked by white dots (*p* < 0.05, two-sided). (D) The first row presents the grand-average topographical maps of P300 mean amplitude for the n-back task. The Pz electrode is marked with a white triangle on the topographical maps. The second row presents the *t*-value topographical maps from the cluster-based permutation test, with significant electrodes marked by white dots (*p* < 0.05/6 = 0.0083, two-sided). The first four *t*-value topographical maps represent comparisons between higher-load conditions and the 0-back condition (i.e., with-load comparisons), while the last two maps depict comparisons between target and nontarget trials in the 2-back and 3-back conditions (i.e., load-free comparisons), respectively. (E and F) The P300 mean amplitudes at the Pz electrode for the oddball task (E) and n-back (F) tasks. The compared condition pairs correspond to those in (D). Note, ns denotes non-significant differences (Bonferroni corrected *p* < 0.05/6 = 0.0083). Each point represents the P300 mean amplitude of an individual participant. Raincloud plots show individual datapoints, probability density distributions, and summary statistics in the boxplots (median as the thick horizontal line, interquartile range within the box, minimum and maximum as the lower and upper whiskers).

Figure 2 displays the P300 evoked by both the oddball and n-back tasks. Consistent with previous studies,^11^^,^^31^^,^^34^ the P300 in both tasks was predominantly distributed over the parietal region (i.e., Pz electrode). Grand-averaged topographical maps revealed similar scalp distributions across the two tasks (Figures 2C and 2D).

For consistency with the MVPA, the ERP univariate analyses were conducted using the same pairs of conditions as those compared in the MVPA. To examine within-task differences, a CBPT was first performed to assess the significance of P300 mean amplitude (Figures 2C and 2D). The results showed that the topographical distribution of the *t*-values for the 2-back target vs. 0-back, 3-back target vs. 0-back, and 2-back target vs. nontarget contrasts showed a parietal/occipital pattern similar to that observed in the oddball vs. standard contrast, consistent with the classical P300 scalp distribution reported in previous studies. In contrast, the 2-back nontarget vs. 0-back and 3-back target vs. nontarget comparisons showed lower similarity of the parietal/occipital pattern relative to that of the oddball task.

Subsequently, to provide electrode-specific statistical comparison, the P300 mean amplitudes at the Pz electrode were extracted and analyzed. For the oddball task, a paired-sample *t* test revealed that the P300 amplitude of the oddball condition (*M* = 3.536 μV, *SD* = 1.622) was significantly larger than that of the standard condition (*M* = 1.250 μV, *SD* = 0.823), *t*(25) = 7.738, *p* < 0.001, Cohen’s *d* = 1.518, 95% CI = [1.114, 1.922] (Figure 2E).

For the n-back task, a series of paired-sample *t*-tests were conducted to compare the P300 mean amplitudes across different conditions (Figure 2F). First, for the with-load comparison, the P300 mean amplitude in the 0-back was compared with that in higher-load conditions. The results showed that neither the 2-back nontarget (*M* = 0.921 μV, *SD* = 0.658; *p* = 0.157) nor the 3-back nontarget (*M* = 0.953 μV, *SD* = 0.601; *p* = 0.066) differed significantly from the 0-back condition (*M* = 0.675 μV, *SD* = 0.512). In contrast, both the 2-back target (*M* = 1.492 μV, *SD* = 0.914; *t*(25) = 4.114, *p* < 0.001, Cohen’s *d* = 0.807, 95% CI [0.403, 1.211]) and the 3-back target (*M* = 1.187 μV, *SD* = 0.874; *t*(25) = 2.844, *p* = 0.009, Cohen’s *d* = 0.558, 95% CI [0.154, 0.962]) elicited significantly larger P300 mean amplitudes than the 0-back condition, although the latter did not survive Bonferroni correction (*p* < 0.05/6 = 0.0083).

Second, for the load-free comparison, P300 mean amplitudes were significantly larger for target than for nontarget trials in the 2-back condition (*t*(25) = 4.135, *p* < 0.001, Cohen’s *d* = 0.811, 95% CI [0.407, 1.215]), whereas no significant difference was observed between target and nontarget trials in the 3-back condition (*p* = 0.081).

Overall, these results indicate that both the oddball and n-back tasks elicited a P300 component predominantly distributed over the parietal/occipital region, with the grand-averaged and statistical topographies exhibiting visual similarity across tasks.

### Multivariate pattern analysis results

#### Oddball task

Before conducting the cross-task decoding analysis, the within-task decoding analysis was performed to ensure that each task elicited distinguishable neural representations.

**Figure 3:**
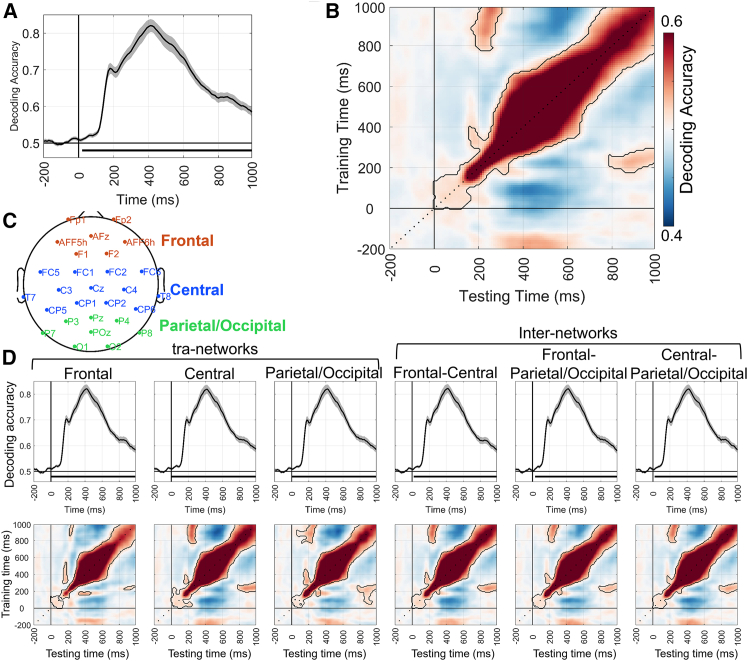
MVPA results for the oddball task (A) The time-by-time decoding analysis results at the global level. The black horizontal line indicates significant decoding intervals (cluster-based permutation test, *p* < 0.05), and the shaded area around the curve represents the standard error of the mean (SEM). (B) The temporal generalization analysis results at the global level. The black curves outline the significant decoding results (cluster-based permutation test, *p* < 0.05). (C) The scalp distribution of electrodes used in this study. Electrodes in the frontal, central, and parietal/occipital regions are color-coded differently. (D) The time-by-time decoding and temporal generalization analysis results at the local level (cluster-based permutation test, *p* < 0.05/6 = 0.0083). Other annotations are the same as in (A and B).

At the global level, a time-by-time decoding analysis was performed on the EEG signals evoked by the oddball and standard conditions. The results revealed that decoding accuracy was significantly above the chance level (*p*_cluster_ < 0.05) from 20 ms to 1000 ms after stimulus onset, with a peak at 410 ms (0.82 ± 0.09; Figure 3A). Moreover, the temporal generalization analysis showed limited generalization around 0–150 ms, followed by broader generalization around 350–700 ms (Figure 3B). Overall, the temporal generalization patterns for the oddball task exhibited a predominantly diagonal pattern, suggesting that the neural representations were mainly characterized by dynamic changes.

At the local level, time-by-time decoding analyses across the six brain networks revealed significantly above-chance accuracy within the following windows: 0–1000 ms, 0–1000 ms, 0–1000 ms, 20–1000 ms, 30–1000 ms, and 20–1000 ms. The peak decoding accuracy occurred consistently at 420 ms (*M* = 0.82, *SD* = 0.09; Figure 3D). Moreover, the time generalization analysis showed that the temporal evolution patterns of neural representations closely resembled those at the global level (Figure 3D).

#### N-back task

**Figure 4:**
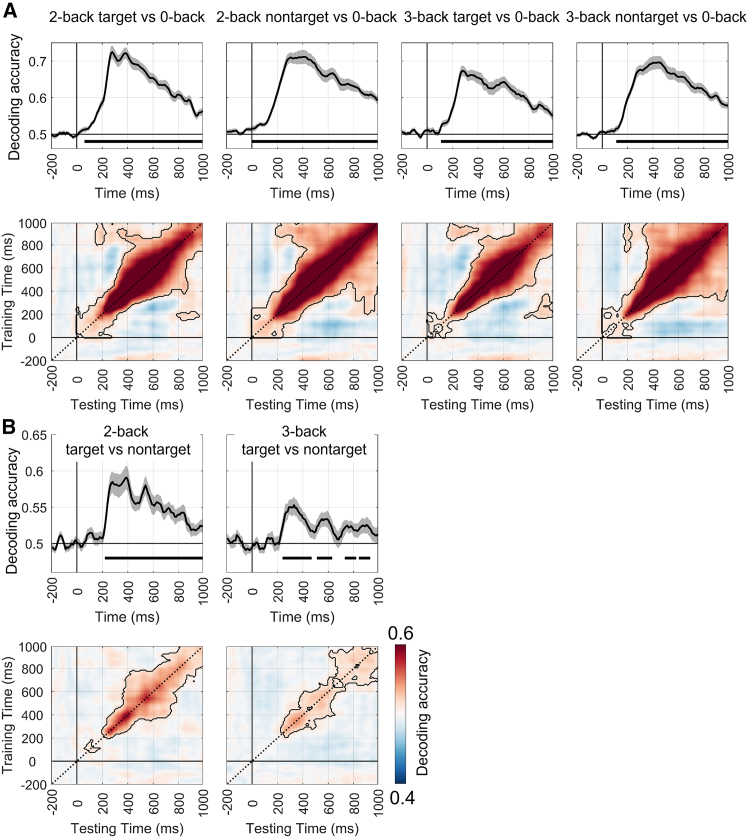
MVPA results for the n-back task (A) Shows the time-by-time decoding and temporal generalization analysis results (cluster-based permutation test, *p* < 0.05) under the with-load condition (i.e., 2-/3-back target/nontarget vs. 0-back). (B) Shows the time-by-time decoding and temporal generalization analysis results under the load-free condition (i.e., 2-/3-back target vs. nontarget). Other annotations are the same as in Figure 3.

For the n-back task, Table 1 presents the time-by-time decoding analysis results at the global level. Compared with the load-free conditions pairs (i.e., 2-/3-back target vs. nontarget), decoding accuracy appeared to be higher under the with-load condition pairs (i.e., 2-/3-back vs. 0-back; Figure 4).

**Table 1:** The time-by-time decoding analysis results for the n-back task

Load	Condition pairs	Significant time range (ms)	Peak time (ms)	Peak acc (*M* ± *SD*)
With-load	2-back target vs. 0-back	60–1000	280	0.72 ± 0.09
	2-back nontarget vs. 0-back	0–1000	400	0.71 ± 0.10
	3-back target vs. 0-back	110–1000	280	0.67 ± 0.07
	3-back nontarget vs. 0-back	110–1000	440	0.70 ± 0.09
Load-free	2-back target vs. 2-back nontarget	220–1000	390	0.59 ± 0.08
	3-back target vs. 3-back nontarget	240–480, 510–640, 730–830, 840–940	330	0.55 ± 0.06

acc, accuracy; *M*, mean; *SD*, standard deviation.

For the temporal generalization analysis, under the with-load condition pairs (i.e., 2-/3-back nontarget vs. 0-back), the temporal generalization results exhibited primarily diagonal patterns before approximately 800 ms, followed by broader generalization patterns (Figure 4A). This suggests a transition from dynamic to more sustained and stable neural representations. In contrast, for the condition pairs 2-/3-back target vs. 0-back, the temporal generalization results showed predominantly diagonal patterns, indicating that the neural representations were mostly transient and dynamic (Figure 4A). Under the load-free condition pairs (i.e., 2-/3-back target vs. nontarget), the temporal generalization results were dominated by diagonal patterns, reflecting primarily dynamic neural representations (Figure 4B).

**Figure 5:**
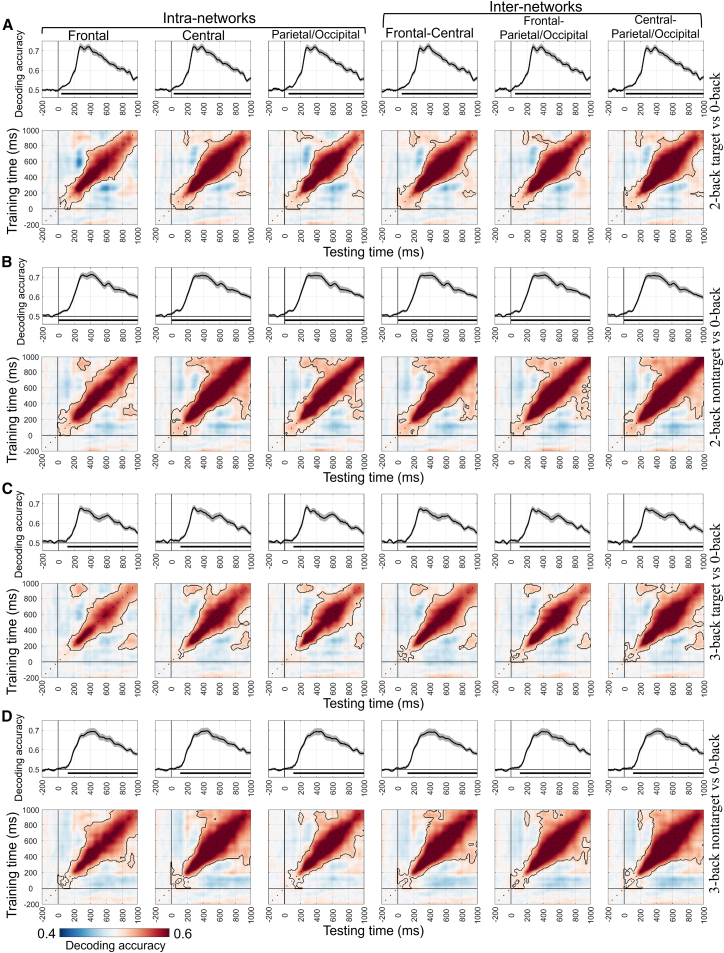
MVPA results for the n-back task in different brain networks under the with-load conditions (A–D) show the time-by-time decoding and temporal generalization analysis results (cluster-based permutation test, *p* < 0.05/6 = 0.0083) for the 2-back target vs. 0-back, 2-back nontarget vs. 0-back, 3-back target vs. 0-back, and 3-back nontarget vs. 0-back condition pairs, respectively.

**Figure 6:**
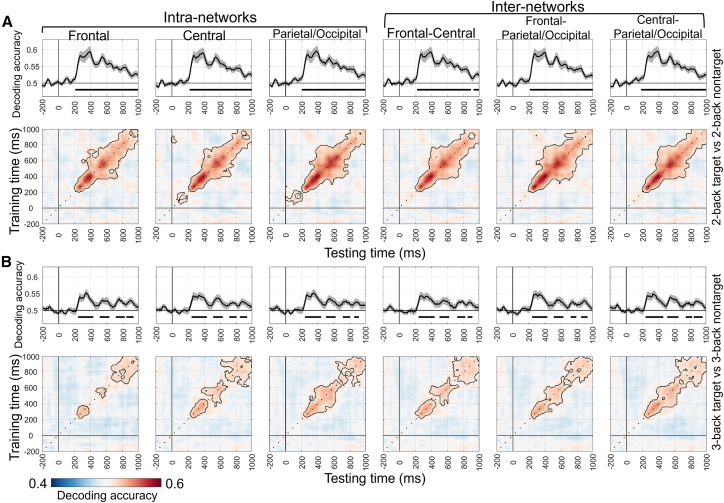
MVPA results for the n-back task in different brain networks under the load-free conditions (A and B) show the time-by-time decoding and temporal generalization analysis results (cluster-based permutation test, *p* < 0.05/6 = 0.0083) for the 2-back target vs. 2-back nontarget and 3-back target vs. 3-back nontarget condition pairs, respectively.

At the local level, this study also reported the MVPA results of six brain networks (see Table 2, Figures 5 and 6). The decoding performance of these brain networks was similar to that observed at the global level.

**Table 2:** The time-by-time decoding analysis results of six networks for the n-back task

Load	Condition pairs	Networks	Significant time range (ms)	Peak time (ms)	Peak acc (*M* ± *SD*)
With-load	2-back target vs. 0-back	Frontal	40–1000	280	0.72 ± 0.09
		Central	30–1000	380	0.72 ± 0.08
		Parietal/Occipital	50–1000	370	0.72 ± 0.07
		Frontal-Central	50–1000	380	0.72 ± 0.08
		Frontal-Parietal/Occipital	50–1000	280	0.72 ± 0.09
		Central-Parietal/Occipital	20–1000	380	0.72 ± 0.08
	2-back nontarget vs. 0-back	Frontal	0–1000	420	0.71 ± 0.09
		Central	0–1000	380	0.71 ± 0.10
		Parietal/Occipital	0–1000	300	0.71 ± 0.07
		Frontal-Central	0–1000	310	0.71 ± 0.07
		Frontal-Parietal/Occipital	0–1000	420	0.71 ± 0.09
		Central-Parietal/Occipital	0–1000	380	0.71 ± 0.10
	3-back target vs. 0-back	Frontal	110–1000	290	0.68 ± 0.07
		Central	110–1000	280	0.68 ± 0.07
		Parietal/Occipital	110–1000	280	0.68 ± 0.07
		Frontal-Central	110–1000	290	0.67 ± 0.07
		Frontal-Parietal/Occipital	110–1000	280	0.67 ± 0.07
		Central-Parietal/Occipital	110–1000	280	0.67 ± 0.07
	3-back nontarget vs. 0-back	Frontal	120–1000	440	0.69 ± 0.09
		Central	110–1000	440	0.70 ± 0.09
		Parietal/Occipital	110–1000	450	0.69 ± 0.09
		Frontal-Central	120–1000	400	0.69 ± 0.09
		Frontal-Parietal/Occipital	110–1000	380	0.69 ± 0.09
		Central-Parietal/Occipital	110–1000	410	0.70 ± 0.09
Load-free	2-back target vs. nontarget	Frontal	210–1000	390	0.59 ± 0.09
		Central	220–1000	380	0.59 ± 0.08
		Parietal/Occipital	200–1000	380	0.60 ± 0.08
		Frontal-central	220–900, 920–1000	390	0.59 ± 0.08
		Frontal-Parietal/Occipital	210–1000	390	0.59 ± 0.08
		Central-Parietal/Occipital	180–1000	380	0.59 ± 0.08
	3-back target vs. nontarget	Frontal	240–440, 510–640, 710–830, 840–940	340	0.55 ± 0.06
		Central	240–440, 510–640, 710–810, 840–940	330	0.54 ± 0.06
		Parietal/Occipital	240–450, 500–630, 710–810, 850–920	350	0.55 ± 0.06
		Frontal-Central	240–450, 500–630, 720–830, 850–920	280	0.54 ± 0.06
		Frontal-Parietal/Occipital	240–440, 510–640, 710–800, 850–940	290	0.55 ± 0.06
		Central-Parietal/Occipital	240–470, 510–660, 740–830, 840–960	340	0.55 ± 0.06

acc, accuracy; *M*, mean; *SD*, standard deviation.

In summary, the within-task MVPA results showed that both oddball and n-back tasks were decodable, particularly during the P300 time window. These results served as a foundation for the subsequent cross-task decoding.

#### Temporal generalization analysis results across tasks

To investigate shared neural mechanisms between the oddball and n-back tasks, a cross-task decoding analysis was performed at both the global and local levels.

**Figure 7:**
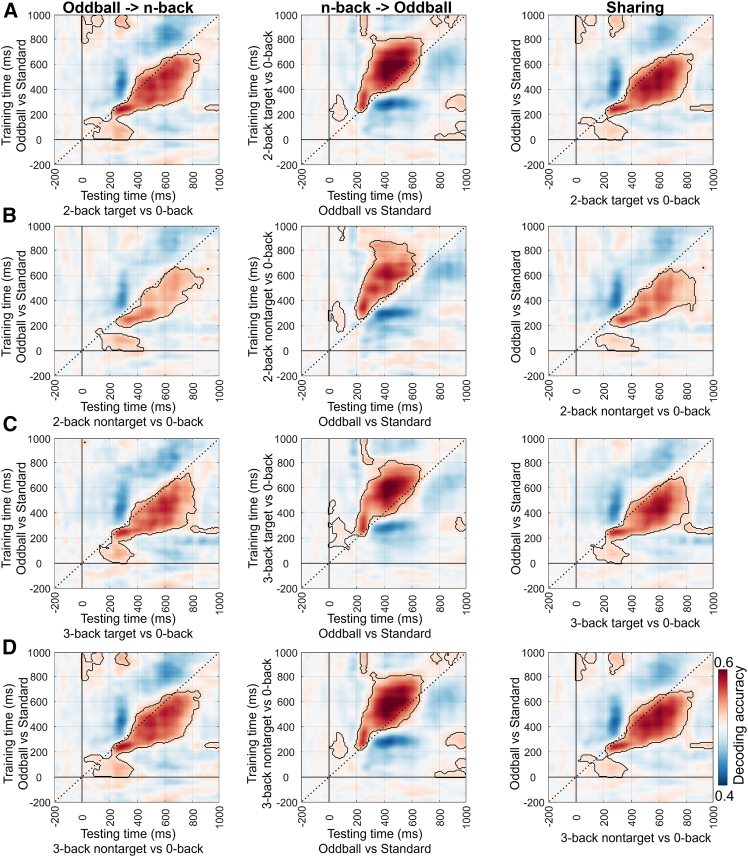
Cross-task decoding results under the with-load condition (2-/3-back target/nontarget vs. 0-back) at the global level (cluster-based permutation test, *p* < 0.05) (A) shows the results where the oddball task (oddball vs. standard) was used as the training set and the n-back task (2-back target vs. 0-back) as the testing set (left); the reversed direction, in which the n-back task was used as the training set and the oddball task as the testing set (middle); and the average of the first two matrices, representing the shared neural representations between the two tasks (right). (B–D), (C), and (D) show the results for the following condition pairs: oddball vs. standard and 2-back nontarget vs. 0-back, oddball vs. standard and 3-back target vs. 0-back, and oddball vs. standard and 3-back nontarget vs. 0-back, respectively. Other annotations are the same as in (A).

Figure 7 presents the results under the with-load condition at the global level. The significant clusters (*p*_cluster_ < 0.05) were mainly located around the P300 time window. Notably, the significant clusters were not symmetrically distributed along the diagonal. Instead, they were predominantly located in the lower right triangle, corresponding to the later time points in the n-back task.

**Figure 8:**
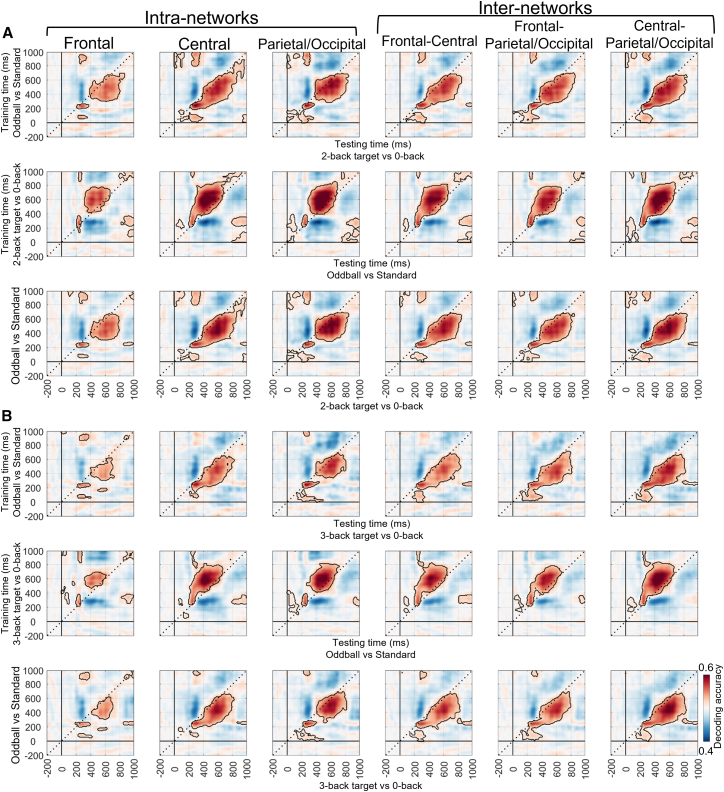
Cross-task decoding results under the with-load condition (2-/3-back target vs. 0-back) at the local level (cluster-based permutation test, *p* < 0.05/6 = 0.0083) (A) The first row shows the results that the oddball task (oddball vs. standard) was used as the training set and the n-back task (2-back target vs. 0-back) as the testing set. The second row presents the reversed direction, with the n-back task used as the training set and the oddball task as the testing set. The third row displays the average of the two matrices above, representing the shared neural representations. Each column represents a specific brain network. (B) Other explanations are the same as in (A), except for the use of the 3-back task.

**Figure 9:**
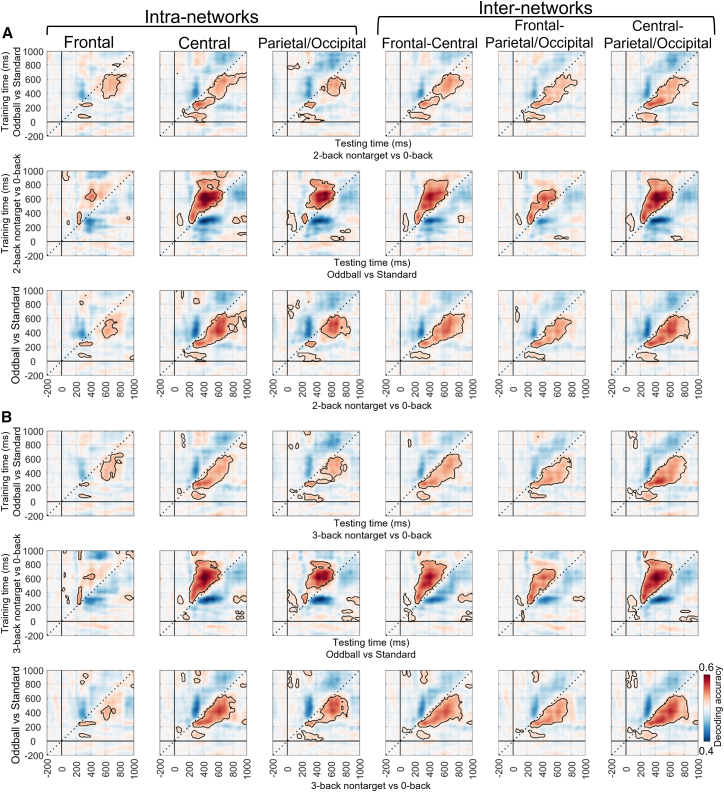
Cross-task decoding results under the with-load condition (2-/3-back nontarget vs. 0-back) at the local level (cluster-based permutation test, *p* < 0.05/6 = 0.0083) (A) The first row shows the results that the oddball task (oddball vs. standard) was used as the training set and the n-back task (2-back nontarget vs. 0-back) as the testing set. The second row presents the reversed direction, with the n-back task used as the training set and the oddball task as the testing set. The third row displays the average of the two matrices above, representing the shared neural representations. Each column represents a specific brain network. (B) Other explanations are the same as in (A), except for the use of the 3-back task.

At the local level, cross-task decoding was performed separately for the six brain networks under the with-load condition. For the oddball (vs. standard) and 2-/3-back target (vs. 0-back) condition pairs, the significant results were largely consistent with those obtained at the global level (Figure 8). It should be noted that some differences were observed relative to the decoding results at the global level. Specifically, decoding performance in the frontal network showed a slight decline under the 2-back target condition (Figure 8A) and a more pronounced decline under the 3-back target condition (Figure 8B). Additionally, for the n-back nontarget conditions, the cross-task decoding results resembled those of the n-back target conditions but showed an overall reduction in decoding performance (Figure 9).

**Figure 10:**
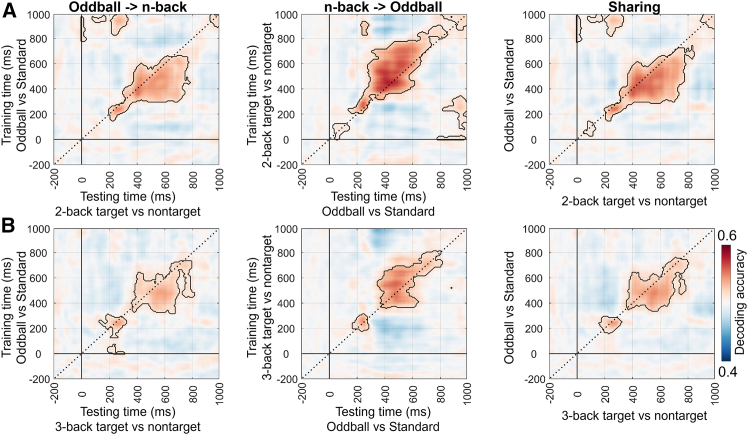
Cross-task decoding results under the load-free condition (2-/3-back target vs. nontarget) at the global level (cluster-based permutation test, *p* < 0.05) (A) shows the results where the oddball task (oddball vs. standard) was used as the training set and the n-back task (2-back target vs. 2-back nontarget) as the testing set (left); the reversed direction, in which the n-back task was used as the training set and the oddball task as the testing set (middle); and the average of the first two matrices, representing the shared neural representations between the two tasks (right). (B) Other explanations are the same as in (A), except for the use of the 3-back task.

**Figure 11:**
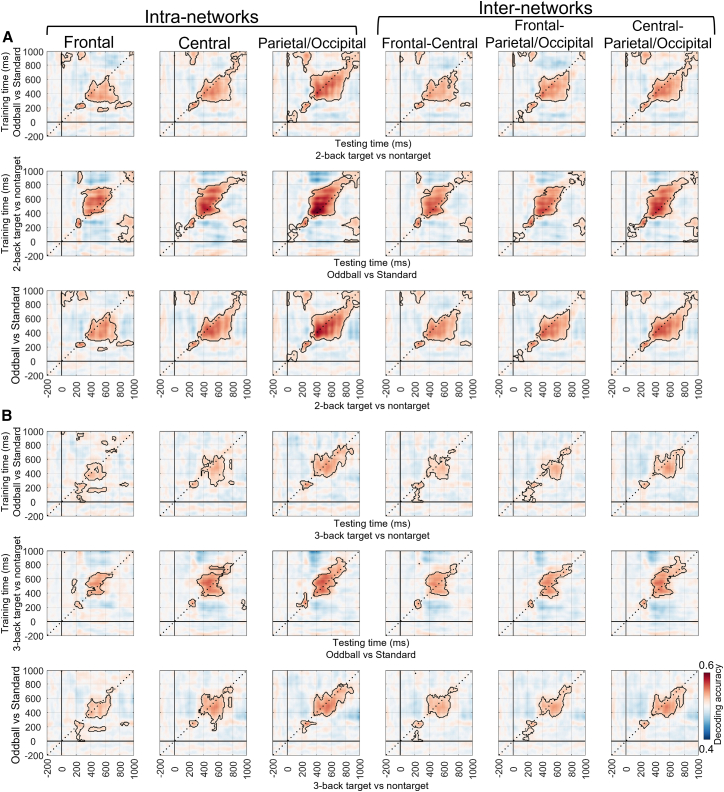
Cross-task decoding results under the load-free condition (2-/3-back target vs. nontarget) at the local level (cluster-based permutation test, *p* < 0.05/6 = 0.0083) (A) The first row shows the results that the oddball task (oddball vs. standard) was used as the training set and the n-back task (2-back target vs. nontarget) as the testing set. The second row presents the reversed direction, with the n-back task used as the training set and the oddball task as the testing set. The third row displays the average of the two matrices above, representing the shared neural representations. Each column represents a specific brain network. (B) Other explanations are the same as in (A), except for the use of the 3-back task.

Moreover, cross-task decoding was also conducted under the load-free conditions of the n-back task (i.e., 2-/3-back target vs. nontarget). The results showed that, at the global level, decoding accuracy remained significant, although overall performance declined (Figure 10), particularly in the 3-back condition (Figure 10B). At the local level, significant decoding accuracy was still observed (Figure 11), although the decline in the 3-back condition was slightly more pronounced, especially in the frontal network (Figure 11B). Overall, these findings support the existence of shared neural representations between the oddball and n-back tasks, particularly around the P300 time window.

**Figure 12:**
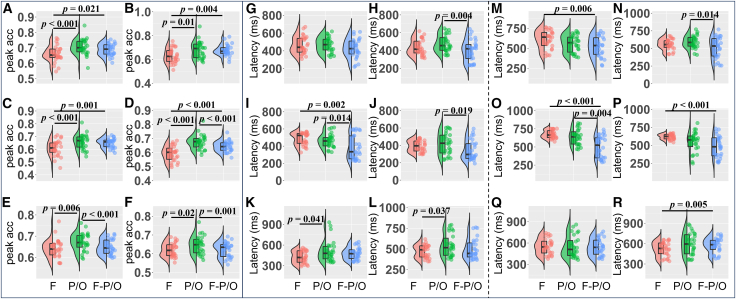
Peak accuracies and latencies of the cross-task decoding across the frontal, parietal/occipital, and frontal-parietal/occipital networks (Bonferroni-adjusted *p* < 0.05) (A–F) illustrate the peak accuracies of the 6 condition pairs: (A) 2-back target vs. 0-back, (B) 3-back target vs. 0-back, (C) 2-back nontarget vs. 0-back, (D) 3-back nontarget vs. 0-back, (E) 2-back target vs. nontarget, and (F) 3-back target vs. nontarget. (G–L) illustrate the latencies of peak accuracies for the oddball task across 6 condition pairs: (G) 2-back target vs. 0-back, (H) 3-back target vs. 0-back, (I) 2-back nontarget vs. 0-back, (J) 3-back nontarget vs. 0-back, (K) 2-back target vs. nontarget, and (L) 3-back target vs. nontarget. (M–R) illustrate the latencies of peak accuracies for the n-back task across 6 condition pairs: (M) 2-back target vs. 0-back, (N) 3-back target vs. 0-back, (O) 2-back nontarget vs. 0-back, (P) 3-back nontarget vs. 0-back, (Q) 2-back target vs. nontarget, and (R) 3-back target vs. nontarget. Note, acc = accuracy; F = frontal; P/O = parietal/occipital; F-P/O = frontal-parietal/occipital.

To further demonstrate that the shared neural mechanism was mainly the P300 rather than the P3a, rmANOVA was used to compare the peak accuracies of the cross-task decoding across the frontal, parietal/occipital, and frontal-parietal/occipital networks under each condition pair, i.e., between the oddball vs. standard and the corresponding 2/3-back (non)target vs. 0-back and 2/3-back target vs. nontarget (Figures 8, 9, and 11). Figures 12A–12F presents the peak accuracies of cross-task decoding across the frontal, parietal/occipital, and frontal-parietal/occipital networks.

First, the with-load comparisons were conducted. For the 2-back target vs. 0-back, the network factor was statistically significant, *F*(1.928, 48.204) = 12.068, *p* < 0.001, η^2^_*p*_ = 0.326. Post hoc analysis showed that peak accuracy in the parietal/occipital network (*M* = 0.705, *SD* = 0.052) was higher than the frontal network (*M* = 0.660, *SD* = 0.054), *t*(25) = 4.535, *p* < 0.001, Cohen’s *d* = 0.956, 95% CI = [0.415, 1.497]. Moreover, the frontal-parietal/occipital network (*M* = 0.689, *SD* = 0.040) was higher than the frontal network, *t*(25) = 2.941, *p* = 0.021, Cohen’s *d* = 0.583, 95% CI = [0.074, 1.092]. No significant difference was found between parietal/occipital and frontal-parietal/occipital networks (*p* > 0.05) (Figure 12A). For the 3-back target vs. 0-back, the network factor was statistically significant, *F*(1.539, 38.468) = 9.610, *p* = 0.001, η^2^_*p*_ = 0.278. Post hoc analysis showed that peak accuracy in the parietal/occipital network (*M* = 0.681, *SD* = 0.072) was higher than the frontal network (*M* = 0.631, *SD* = 0.062), *t*(25) = 3.261, *p* = 0.010, Cohen’s *d* = 0.773, 95% CI = [0.165, 1.382]. Moreover, the frontal-parietal/occipital network (*M* = 0.677, *SD* = 0.048) was also higher than the frontal network, *t*(25) = 3.589, *p* = 0.004, Cohen’s *d* = 0.712, 95% CI = [0.203, 1.221]. No significant difference was found between the parietal/occipital and frontal-parietal/occipital networks (*p* > 0.05) (Figure 12B). For the 2-back nontarget vs. 0-back, the network factor was statistically significant, *F*(1.792, 44.805) = 13.234, *p* < 0.001, η^2^_*p*_ = 0.346. Post hoc analysis showed that peak accuracy in the parietal/occipital network (*M* = 0.658, *SD* = 0.059) was higher than the frontal network (*M* = 0.609, *SD* = 0.063), *t*(25) = 4.207, *p* < 0.001, Cohen’s *d* = 0.948, 95% CI = [0.370, 1.527]. Moreover, the frontal-parietal/occipital network (*M* = 0.649, *SD* = 0.037) was also higher than the frontal network, *t*(25) = 4.068, *p* = 0.001, Cohen’s *d* = 0.773, 95% CI = [0.285, 1.260]. No significant difference was found between the parietal/occipital and frontal-parietal/occipital networks (*p* > 0.05) (Figure 12C). For the 3-back nontarget vs. 0-back, the network factor was statistically significant, *F*(1.438, 35.960) = 32.804, *p* < 0.001, η^2^_*p*_ = 0.568. Post hoc analysis showed that peak accuracy in the parietal/occipital network (*M* = 0.674, *SD* = 0.052) was higher than the frontal network (*M* = 0.592, *SD* = 0.054; *t*(25) = 6.533, *p* < 0.001, Cohen’s *d* = 1.573, 95% CI = [0.955, 2.190]) and the frontal-parietal/occipital network (*M* = 0.643, *SD* = 0.040; *t*(25) = 4.608, *p* < 0.001, Cohen’s *d* = 0.593, 95% CI = [0.263, 0.923]). Moreover, the frontal-parietal/occipital network was also higher than the frontal network, *t*(25) = 4.846, *p* < 0.001, Cohen’s *d* = 0.980, 95% CI = [0.461, 1.499] (Figure 12D).

Second, the load-free comparisons were conducted. For the 2-back target vs. nontarget, the network factor was statistically significant, *F*(1.542, 38.560) = 8.037, *p* = 0.003, η^2^_*p*_ = 0.243. Post hoc analysis showed that peak accuracy in the parietal/occipital network (*M* = 0.673, *SD* = 0.043) was higher than the frontal (*M* = 0.643, *SD* = 0.044), *t*(25) = 3.472, *p* = 0.006, Cohen’s *d* = 0.746, 95% CI = [0.195, 1.297] and the frontal-parietal/occipital networks (*M* = 0.649, *SD* = 0.036), *t*(25) = 4.417, *p* < 0.001, Cohen’s *d* = 0.588, 95% CI = [0.247, 0.930]. No significant difference was found between the frontal-parietal/occipital and frontal networks (*p* > 0.05) (Figure 12E). For the 3-back target vs. nontarget, the network factor was significant, *F*(1.529, 38.229) = 7.550, *p* = 0.004, η^2^_*p*_ = 0.232. Post hoc analysis showed that peak accuracy in the parietal/occipital network (*M* = 0.646, *SD* = 0.049) was higher than the frontal network (*M* = 0.620, *SD* = 0.037), *t*(25) = 2.959, *p* = 0.020, Cohen’s *d* = 0.687, 95% CI = [0.091, 1.283] and the frontal-parietal/occipital network (*M* = 0.623, *SD* = 0.040), *t*(25) = 4.686, *p* < 0.001, Cohen’s *d* = 0.628, 95% CI = [0.284, 0.973]. No significant difference was found between the frontal-parietal/occipital and frontal networks (*p* > 0.05) (Figure 12F). Taken together, these results demonstrate that cross-task decoding performance consistently peaked in the parietal/occipital network across all comparisons, whereas the frontal network exhibited lower accuracy. This pattern confirms that the shared neural mechanism underlying the oddball and n-back tasks is primarily associated with the parietal/occipital P300 rather than the frontal P3a.

Moreover, to further examine the temporal characteristics of shared neural mechanisms between the oddball and n-back tasks, we compared the latency of peak decoding accuracies across the frontal, parietal/occipital, and frontal-parietal/occipital networks. Figures 12G–L) and (M-R) present the latencies of peak accuracies for the oddball task and the n-back task, respectively.

##### Oddball task

First, the with-load comparisons were conducted. For the 2-back target vs. 0-back, the network factor was not statistically significant, *F*(1.937, 48.418) = 3.202, *p* = 0.051, η^2^_*p*_ = 0.114 (Figure 12G). For the 3-back target vs. 0-back, the network factor was statistically significant, *F*(1.990, 49.744) = 6.536, *p* = 0.003, η^2^_*p*_ = 0.207. Post hoc analysis showed that latency of peak accuracy in the frontal-parietal/occipital network (*M* = 401.154 ms, *SD* = 122.714) was shorter than the parietal/occipital network (*M* = 477.308 ms, *SD* = 94.554), *t*(25) = −3.590, *p* = 0.004, Cohen’s *d* = −0.707, 95% CI = [−1.212, −0.202]. No further effect reached significance (*p*s > 0.05) (Figure 12H). For the 2-back nontarget vs. 0-back, the network factor was statistically significant, *F*(1.634, 40.844) = 10.346, *p* < 0.001, η^2^_*p*_ = 0.293. Post hoc analysis showed that latency of peak accuracy in the frontal-parietal/occipital network (*M* = 384.231 ms, *SD* = 141.285) was shorter than the frontal network (*M* = 485.000 ms, *SD* = 72.346), *t*(25) = −3.826, *p* = 0.002, Cohen’s *d* = −0.845, 95% CI = [−1.411, −0.278] and the parietal/occipital network (*M* = 464.231 ms, *SD* = 79.306), *t*(25) = −3.115, *p* = 0.014, Cohen’s *d* = −0.671, 95% CI = [−1.223, −0.118]. No further effect reached significance (*p*s > 0.05) (Figure 12I). For the 3-back nontarget vs. 0-back, the network factor was statistically significant, *F*(1.940, 48.488) = 5.240, *p* = 0.009, η^2^_*p*_ = 0.173. Post hoc analysis showed that latency of peak accuracy in the frontal-parietal/occipital network (*M* = 346.154 ms, *SD* = 116.347) was shorter than the parietal/occipital network (*M* = 418.846 ms, *SD* = 124.878), *t*(25) = −2.979, *p* = 0.019, Cohen’s *d* = −0.628, 95% CI = [−1.170, −0.087]. No further effect reached significance (*p*s > 0.05) (Figure 12J).

Second, the load-free comparisons were conducted. For the 2-back target vs. nontarget, the network factor was statistically significant, *F*(1.579, 39.475) = 4.551, *p* = 0.024, η^2^_*p*_ = 0.154. Post hoc analysis showed that the latency of peak accuracy in the parietal/occipital network (*M* = 513.846 ms, *SD* = 159.200) was longer than the frontal network (*M* = 425.385 ms, *SD* = 88.237), *t*(25) = 2.652, *p* = 0.041, Cohen’s *d* = 0.590, 95% CI = [0.019, 1.161]. No further effect reached significance (*p*s > 0.05) (Figure 12K). For the 3-back target vs. nontarget, the network factor was statistically significant, *F*(1.719, 42.962) = 4.486, *p* = 0.021, η^2^_*p*_ = 0.152. Post hoc analysis showed that the latency of peak accuracy in the parietal/occipital network (*M* = 541.923 ms, *SD* = 134.581) was longer than the frontal network (*M* = 470.385 ms, *SD* = 78.967), *t*(25) = 2.695, *p* = 0.037, Cohen’s *d* = 0.583, 95% CI = [0.028, 1.139]. No further effect reached significance (*p*s > 0.05) (Figure 12L).

##### N-back task

First, the with-load comparisons were conducted. For the 2-back target vs. 0-back, the network factor was statistically significant, *F*(1.744, 43.601) = 8.129, *p* = 0.002, η^2^_*p*_ = 0.245. Post hoc analysis showed that the latency of peak accuracy in the frontal-parietal/occipital network (*M* = 520.385 ms, *SD* = 127.984) was significantly shorter than the frontal network (*M* = 608.846 ms, *SD* = 116.080), *t*(25) = −3.441, *p* = 0.006, Cohen’s *d* = −0.788, 95% CI = [−1.375, −0.200]. No further effect reached significance (*p*s > 0.05) (Figure 12M). For the 3-back target vs. 0-back, the network factor was statistically significant, *F*(1.673, 41.830) = 5.699, *p* = 0.009, η^2^_*p*_ = 0.186. Post hoc analysis showed that latency of peak accuracy in the frontal-parietal/occipital network (*M* = 511.154 ms, *SD* = 151.534) was shorter than the parietal/occipital network (*M* = 590.769 ms, *SD* = 90.241), *t*(25) = −3.107, *p* = 0.014, Cohen’s *d* = −0.659, 95% CI = [−1.203, −0.115]. No further effect reached significance (*p*s > 0.05) (Figure 12N). For the 2-back nontarget vs. 0-back, the network factor was statistically significant, *F*(1.654, 41.359) = 16.523, *p* < 0.001, η^2^_*p*_ = 0.398. Post hoc analysis showed that the latency of peak accuracy in the frontal-parietal/occipital network (*M* = 503.846 ms, *SD* = 152.868) was shorter than the frontal network (*M* = 672.692 ms, *SD* = 55.538), *t*(25) = −5.449, *p* < 0.001, Cohen’s *d* = −1.071, 95% CI = [−1.575, −0.566]. Moreover, latency of peak accuracy in the frontal-parietal/occipital network (*M* = 503.846 ms, *SD* = 152.868) was shorter than the parietal/occipital network (*M* = 636.539 ms, *SD* = 109.103), *t*(25) = −3.633, *p* = 0.004, Cohen’s *d* = −0.841, 95% CI = [−1.435, −0.247]. No further effect reached significance (*p*s > 0.05) (Figure 12O). For the 3-back nontarget vs. 0-back, the network factor was statistically significant, *F*(1.999, 49.981) = 11.617, *p* < 0.001, η^2^_*p*_ = 0.317. Post hoc analysis showed that latency of peak accuracy in the frontal-parietal/occipital network (*M* = 479.615 ms, *SD* = 150.293) was shorter than the frontal network (*M* = 619.231 ms, *SD* = 36.652), *t*(25) = −4.844, *p* < 0.001, Cohen’s *d* = −0.945, 95% CI = [−1.446, −0.445]. No further effect reached significance (*p*s > 0.05) (Figure 12P).

Second, the load-free comparisons were conducted. For the 2-back target vs. nontarget, the network factor was not statistically significant, *F*(1.757, 43.930) = 0.071, *p* = 0.911, η^2^_*p*_ = 0.003 (Figure 12Q). For the 3-back target vs. nontarget, the network factor was statistically significant, *F*(1.290, 32.256) = 3.966, *p* = 0.045, η^2^_*p*_ = 0.137. Post hoc analysis showed that the latency of peak accuracy in the frontal-parietal/occipital network (*M* = 576.154 ms, *SD* = 107.818) was longer than the frontal network (*M* = 522.692 ms, *SD* = 106.341), *t*(25) = 3.529, *p* = 0.005, Cohen’s *d* = 0.390, 95% CI = [0.107, 0.674]. No further effect reached significance (*p*s > 0.05) (Figure 12R). In sum, the temporal profiles of cross-task decoding provided converging evidence, showing that the parietal/occipital network exhibited longer latencies of peak accuracy than the frontal network, particularly in the load-free comparisons (2/3-back target vs. nontarget), further supporting the predominance of the P300 as the shared neural mechanism.

## Discussion

This study aimed to investigate whether the P300 serves as a shared neural mechanism between the detection of oddball targets and WM updating. Using temporal generalization MVPA, this study conducted cross-task decoding on EEG patterns elicited by the oddball and n-back tasks at both global and local levels. The results showed that cross-task decoding performance was significantly above chance level, predominantly within the classical P300 time window. Moreover, decoding accuracy in the parietal/occipital network was higher than in the frontal network, indicating that the shared neural mechanism reflected the P300 rather than the P3a. Together, these findings suggest that the P300 is largely a shared neural mechanism between the detection of oddball targets and WM updating. From the perspective of multivariate neural representations, this study provides tentative evidence for the WM updating theory of P300.

### P300 as a shared neural mechanism underlying the detection of oddball targets and working memory updating

The P300 is typically elicited by oddballs. The prevalent interpretation of this ERP component is the context (or WM) updating theory.^7^ Specifically, after the initial encoding of sensory input, attentional resources are allocated to comparing newly presented stimuli with those retained in WM. If the incoming stimulus matches a previously held representation, only the early sensory-related ERP components (e.g., N100, P200, and N200) are observed. However, when a mismatch is detected, the brain updates its mental representation model based on the novel information, resulting in a larger amplitude of P300.^1^ Since its proposal, however, this theory has only been examined in a limited number of studies using traditional univariate analyses.^31^^,^^43^^,^^44^ Based on the WM updating theory of P300, the present study hypothesized that P300 is a shared neural mechanism underlying the detection of oddball targets and WM updating. To test this hypothesis, this study combined MVPA with EEG data to directly examine whether there is cross-task neural overlap between the EEG patterns elicited by the oddball and n-back tasks. At the global level, this study found that EEG patterns from the oddball and n-back tasks (2-/3-back target/nontarget vs. 0-back) could be mutually decoded across tasks, with significant effects primarily occurring around the P300 time window. Notably, these effects persisted even when controlling for WM load (i.e., 2-/3-back target vs. nontarget). These findings are consistent with the expectation that there is neural similarity or overlap between the P300 elicited by the two tasks. This suggests that the P300 is largely a shared neural mechanism between the detection of oddball targets and WM updating.

Furthermore, this study divided the scalp electrodes into six brain networks and investigated the shared neural mechanism at the local level. The results showed that the strongest neural overlap effect was located in the posterior parietal/occipital network, consistent with the classical P300 topography. This supports the idea that the shared mechanism underlying the detection of oddball targets and WM updating is mainly the P300 subcomponent, rather than the P3a. P3a, often referred to as “novelty P300”, is predominantly distributed over the frontal region.^3^^,^^59^ Collectively, these findings provide initial evidence supporting P300 as a shared neural mechanism between the detection of oddball targets and WM updating to a substantial degree, which is consistent with the WM updating theory of the P300.

These findings may not be surprising for two main reasons. First, theoretically, the WM updating theory posits that the P300 is not merely a tactical response to a single stimulus or task, but rather reflects a more strategic process (i.e., constantly updating and maintaining a mental model of the environment based on current stimulus information). In the context of a constantly changing environment, the mismatch between new input and the existing model triggers the P300, therefore facilitating model revision.^60^ Consequently, both the detection of rare events in the oddball paradigm and the continuous updating of WM in response to target stimuli in the n-back task may rely on this shared WM updating mechanism, thereby exhibiting highly similar neural representation patterns. Second, the P300 elicited by the oddball and n-back tasks exhibits remarkably similar scalp topographies, which is consistently observed both in the grand-averaged topographies in this study (Figure 2) and in previous literature.^3^^,^^31^^,^^61^^,^^62^ However, both lines of speculation were based solely on univariate analyses. There is strong evidence that the activation of the same brain regions across different tasks does not necessarily indicate the presence of a shared neural mechanism.^4^^,^^45^^,^^46^^,^^47^ This is because even if two tasks activate highly overlapping brain regions at the univariate level, these regions may still contain functionally independent neural populations. This phenomenon has been strongly confirmed by MVPA, which is more sensitive than traditional univariate analyses.^46^^,^^47^^,^^63^ For example, for some condition pairs, cross-task decoding yielded significant results even when the corresponding univariate analyses did not reach the significance threshold (Figures 2D and 2F). Importantly, some researchers have argued that, compared with univariate analyses, MVPA provides a principled and standardized way to quantify shared neural mechanisms across tasks by examining multivariate neural representation patterns.^4^ At the global level, the present study used MVPA to confirm that the P300 is largely a shared neural mechanism across tasks at the single-trial level within each individual. That is, the multivariate pattern of the oddball-P300 in each participant corresponded strictly to that of their own n-back-P300, rather than a grand-averaged result.

At the local level, this study found that the cross-task decoding performance of the parietal/occipital network was significantly higher than that of the frontal network. This was expected, as the primary shared neural mechanism was the P300 rather than the P3a. In addition, the frontal-parietal/occipital network also outperformed the frontal network but showed comparable decoding accuracy to the parietal/occipital network, particularly under the with-load conditions (Figures 12A–12D). Intuitively, one might expect that the frontal-parietal/occipital network would yield superior decoding performance due to its larger number of electrodes and thus higher feature dimensionality. However, the present results did not show such an advantage. A plausible explanation lies in the network dynamics underlying P300 generation. According to the two-process model, the P300 arises from functional networks linking the frontal-temporal and frontal-parietal regions.^1^ Specifically, during the P300, signals originating from the frontal region are transmitted to the posterior region.^1^^,^^64^ The frontal region may exert top-down modulation over parietal regions.^65^ This notion of the P300 as a product of distributed network interactions has been supported by converging evidence from fMRI, intracranial recordings, and EEG source localization studies.^66^ Consistent with this framework, comparable decoding performance between the frontal-parietal/occipital and parietal/occipital networks may suggest that parietal/occipital activity already contains information patterns that are closely associated with, or partially reflect, frontal activity. In conclusion, these findings of this study suggest that P300 is largely a shared neural mechanism underlying the detection of oddball targets and WM updating at both the global and local levels.

Importantly, these findings are consistent with the theoretical framework of the multiple-demand (MD) system proposed by Duncan.^67^ The MD system comprises core regions primarily located in the frontal and parietal cortices. These regions constitute a critical network that supports a wide range of high-level cognitive tasks by flexibly coordinating “mental programs”. Furthermore, the MD system is widely regarded as a domain-general mechanism across various cognitive domains. A recent electrophysiological study that combined MVPA with aperiodic activity and neural oscillations (i.e., periodic activity) provided empirical support for the domain-general function of the MD system.^57^ The present study extends this framework to the ERPs domain, suggesting that the P300 may serve as a potential electrophysiological marker of the MD system. Nevertheless, this interpretation should be viewed with caution as the hypothesis that P300 is associated with the frontal-parietal/occipital network requires further verification. Theoretical and empirical studies suggest that the transmission of signals from the frontal to the parietal/occipital may be biophysically mediated through phase-synchronized oscillatory activity,^65^^,^^68^^,^^69^ while our inference is based on sensor-level multivariate patterns.

The present findings are also consistent with the theory of neural reuse. This theory proposes that the repurposing of neural circuits is a core organizational principle underlying various cognitive functions. According to this theory, neural circuits originally developed for one function may be reused, recycled, or redeployed for different purposes throughout evolution or typical development, often without losing their original function.^70^^,^^71^^,^^72^ This hypothesis has been supported by numerous fMRI studies, which revealed shared neural mechanisms in certain brain regions or networks across different cognitive domains such as finger recognition (or representation) and numerical representation in the left precentral gyrus and left angular gyrus,^73^^,^^74^ creativity and WM in the default mode network and executive control network,^75^^,^^76^^,^^77^^,^^78^ intelligence and creativity in the frontal cortex,^79^ and curiosity and creativity in the default mode network, salience network, and executive control network.^80^ In addition to these fMRI studies, similar findings have also been reported in the field of electrophysiological research, such as WM, switching, and multi-source interference,^57^ perception and imagery,^55^^,^^56^ and the detection of oddball targets and syntactic violations.^4^ Together, this study, in conjunction with preceding research, has expanded the application scope of MD network and neural reuse theories from the perspective of electrophysiology.

It is important to note that the temporal range of neural overlap observed in this study was not symmetrically distributed along the diagonal. Compared to the oddball task, the neural overlap occurred at a relatively later stage in the n-back task, primarily within the lower triangular range. This pattern is expected. The oddball tasks require responses to rare and standard stimuli, primarily involving processes such as identification and maintenance. In contrast, the cognitive processes of the n-back task include not only recognition and maintenance but also updating, inhibition of distractors, and other processes.^14^^,^^81^ Compared to the oddball task, the n-back task is a more complex paradigm. Moreover, P300 latency, an index of processing speed, generally increases with task difficulty.^59^ This finding is supported by behavioral reaction time results. Previous study has reported that the reaction time for 2-back (761 ± 201 ms) and 3-back (833 ± 195 ms) is longer than that for the oddball stimuli (425 ± 53 ms).^58^ Therefore, the time range of neural overlap is manifested as a relative delay in the n-back task. This result not only corroborates prior findings that P300 latency reflects decision-related processing^7^^,^^59^^,^^82^ but also supports the methodological advantage of the temporal generalization cross-task decoding approach used in this study.

However, an alternative interpretation of our findings in terms of target detection cannot be completely ruled out. For the oddball task, previous studies have suggested that the oddball-P300 may represent a domain-general brain response to salience.^3^^,^^4^ By contrast, the n-back task engages more complex cognitive processes. Logically, the 0-back task primarily requires matching—comparing the current stimulus with information in WM—and is thus functionally similar to the oddball task. The 1-, 2-, and 3-back tasks additionally require continuous updating of stored representations.^21^^,^^81^^,^^83^^,^^84^ Therefore, the observed neural overlap during the P300 time window may suggest that P300 is largely a shared neural mechanism between the detection of oddball targets and WM updating, consistent with the context updating interpretation of the oddball-P300.^7^^,^^31^ Meanwhile, we acknowledge that the n-back task may also involve a target detection process, rather than reflecting purely WM updating. Importantly, the time window identified in our results corresponds primarily to the P300—neither the earlier N2pc, typically associated with selection and recognition, nor the later negative slow waves associated with the maintenance of encoded items.^11^ Thus, while we cannot exclude contributions from target detection, the timing and functional characteristics of the observed activity suggest that WM updating remains the most plausible mechanism of the oddball-P300. Future studies could employ paradigms such as the reference-back task^43^^,^^44^ to better isolate WM updating and more precisely characterize its relationship with the P300.

The present findings do not reveal the nature of the P300 as a cross-task shared neural mechanism. One possible explanation is that it may be a result of locus coeruleus (LC) neuron firing,^85^^,^^86^ which leads to a phasic increase in noradrenaline release across the cortex.^85^^,^^87^^,^^88^^,^^89^ According to this hypothesis, the amplitude of the P300 is modulated by stimulus categorization. Events are classified as either matching or not matching a mental representation of a stimulus or category (i.e., the target) stored in memory. Therefore, when an event is identified as matching the target representation, it elicits a larger P300 amplitude than one that is not. In addition, only the LC-P300 hypothesis proposes that the P300 reflects a selective response of the locus coeruleus-norepinephrine (LC-NE) system to task-relevant stimuli. This response is considered part of the attentional mechanism and may be indirectly related to WM updating, but does not reflect the updating process itself.^90^ Moreover, the P300 is also modulated by dopaminergic activity.^1^^,^^91^ Dopamine is synthesized in subcortical regions such as the ventral tegmental area (VTA) and substantia nigra, and is projected through various dopaminergic pathways to numerous brain areas, including the striatum and the prefrontal cortex.^92^ These two regions play important roles in WM updating.^93^ All of these mechanisms may account for the generation of the P300. Future research should delineate the specific brain regions and neurotransmitter systems and their interactions that underlie P300 generation.

### The potential of multivariate pattern analysis to detect neural overlap between different cognitive processes

With advances in cognitive neuroscience, traditional univariate analysis for EEG/MEG data is no longer sufficient to fully characterize brain activity. There is an urgent need for more fine-grained and sensitive approaches to identify and analyze neural activity patterns. MVPA is one such method, which has been successfully applied in functional neuroimaging to decode brain states that remain imperceptible to univariate approaches.^51^^,^^54^ Compared to univariate approaches, MVPA has several advantages. First, MVPA is a reference-free method and is largely independent of specific filter settings. It can detect neural signals containing discriminative information (i.e., specific patterns of neural activity), irrespective of the electrodes on which the effect emerges. Second, MVPA circumvents the need for high inter-individual consistency in ERP amplitudes. Instead, it relies more on the within-subject consistency of event-related EEG signals (i.e., the stable cognitive processing mechanisms within individuals). This characteristic results in a relatively consistent distribution of trial samples from each condition in neural representation space (e.g., oddball stimuli are distributed around spatial location 1, while standard stimuli are distributed around spatial location 2). In contrast, univariate analysis results are often less stable due to individual heterogeneity. Therefore, MVPA is more robust to inter-individual differences in neural activation patterns. Third, even simple cognitive processes are represented in the brain in a complex and network-based manner rather than by the activation of isolated neural units. By considering the relationships between multiple electrodes (i.e., neural activity patterns), MVPA can more comprehensively capture the underlying neural mechanisms of cognitive processes. Fourth, MVPA enables the examination of the temporal evolution of neural representation patterns through TGA, which allows researchers to infer whether a cognitive process persists or recurs over time. It has also been applied across different tasks, as demonstrated in the present study. When a classifier is trained on one task and tested on another, it can reveal the time points at which the cognitive processes underlying the two tasks show unity (i.e., successful classification) or diversity (i.e., failed classification). Using this approach, the present study found that an MVPA classifier trained on the oddball task could successfully classify condition pairs in the n-back task (and vice versa), clearly illustrating the significant time windows for classification and the patterns of temporal delay in the n-back task.

Beyond the investigation of the shared neural mechanisms between the oddball and n-back tasks in this study, MVPA holds vast potential for addressing a wide range of other questions in cognitive neuroscience. Future research can integrate MVPA with EEG to assess the degree of mutual decoding between subcomponents of executive function (i.e., updating, inhibition, and shifting). This approach would elucidate the degree of unity and diversity among these subcomponents and identify the specific ERP components (or time windows) in which such unity and diversity occur. These executive function subcomponents are closely related to the cognitive and neural processes underlying the oddball and n-back tasks.^28^^,^^47^ Investigating these subcomponents could deepen our understanding of the present findings. Furthermore, MVPA, at least as an important complement to univariate analysis, can help address the open questions regarding the specificity of well-established ERP components, including not only the P300 but also N170, N200, N400, P600, and LPP. This can facilitate the refinement of the functional boundaries of these ERP components.

It is important to clarify that the current study focused on whether the P300 observed at parietal/occipital electrodes functions as a shared neural mechanism across tasks. Analyses at the sensor level cannot definitively determine whether two scalp effects originate from identical neural sources, due to the inherent inverse problem of EEG: a single scalp potential distribution can result from infinitely many possible source configurations. While hemodynamic imaging methods (e.g., fMRI) may help to address this issue, the present question concerns an EEG-defined phenomenon—the scalp-recorded P300—and therefore necessarily requires EEG-based analyses. Therefore, we did not attempt to address the question of shared neural mechanisms by comparing the anatomical sources of the P300 between tasks. Rather, our approach focused on comparing the EEG-based multivariate patterns at the sensor level.

From the perspective of multivariate neural representation, this study supports the view that the P300 is a shared neural mechanism between the detection of oddball targets and WM updating, offering both theoretical and practical implications. Theoretically, the WM updating theory of P300 has long lacked direct empirical data. By applying MVPA across tasks, this study provides preliminary but compelling evidence in favor of that theory. Furthermore, by examining multivariate EEG patterns of the P300, this study provides direct and explicit evidence linking two traditionally parallel cognitive domains (i.e., oddball and WM). The present study found that the P300 elicited by the detection of oddball targets and WM updating not only overlap in terms of univariate brain region activation but also exhibit a strict overlap in multivariate neural representation patterns. This finding not only advances the integration and connection of the two cognitive processing domains but also supports the notion that P300 may function as a domain-general mechanism for multiple cognitive processing demands. Practically, the cross-task MVPA strategy and findings of the present study offer important implications for research on P300 in both developmental and psychopathological fields. In the field of developmental and aging research, beyond examining traditional indicators such as P300 amplitude and latency,^11^^,^^13^^,^^94^ cross-task MVPA can be used to track age-related changes in the multivariate patterns of P300 across individuals. This approach may provide novel perspectives for understanding cognitive and neural development. As a single cognitive process may differentiate into distinct (sub)processes with increasing age,^95^ it is plausible that the degree of shared neural mechanisms across tasks declines with age. Moreover, in a variety of psychiatric and neurological conditions, including attention deficit hyperactivity disorder (ADHD), schizophrenia, depression, high risk for psychosis, obsessive-compulsive disorder (OCD), and Alzheimer’s disease, abnormalities in P300 amplitude and latency have been widely reported.^16^^,^^17^^,^^96^^,^^97^^,^^98^^,^^99^^,^^100^ The cross-task MVPA could further evaluate the shared and task-specific impairments in neural representations between the oddball and WM tasks in these populations. This approach has the potential not only to deepen our understanding of the pathogenesis of mental disorders but also to provide novel insights into the development of targeted interventions.

In sum, this study applies the temporal generalization MVPA to EEG data elicited by oddball and n-back tasks. The results revealed that EEG signals elicited by these two tasks are mutually decodable. The key finding is that the P300 is largely a shared neural mechanism between the detection of oddball targets and WM updating at both the global and local levels. From the perspective of neural representations, these findings strongly support the context updating theory of the P300, deepen our understanding of its cognitive and neural mechanisms, and highlight the potential of MVPA in addressing analogous research questions. These findings may offer novel insights into P300-based research in the contexts of typical development (or aging) and psychopathology.

### Limitations of the study

This study also has several limitations. First, although the sample size was sufficient for group-level statistical analysis, this study had limited power to explore individual differences. Future research could recruit a larger and more diverse participant pool to examine how individual differences, such as fluid intelligence, attention allocation strategies, age, and clinical neurological or psychiatric disorders, influence the cross-task shared patterns of the P300. Second, the spatial resolution of the EEG limits the precise source localization of the P300. Future studies could use combined EEG-fMRI techniques to improve spatial specificity and further refine the anatomical localization of the P300 as a shared neural mechanism. Third, this study employed a linear SVM classifier to ensure computational efficiency and clear interpretability. However, the linear nature of SVM limits its ability to capture complex nonlinear mappings between neural activity and cognitive functions.^101^^,^^102^^,^^103^ In contrast, deep learning-based decoding approaches can overcome these limitations by modeling nonlinear relationships and capturing latent neural features that may not be detected by linear classifiers.^104^^,^^105^^,^^106^^,^^107^ Future research could integrate these nonlinear classifiers into cross-task MVPA frameworks to better elucidate how the P300 serves as a shared neural mechanism. Fourth, the present study used different types of visual stimuli in the two tasks (“O/X” symbols in the oddball task and digits in the n-back task). Although these low-level perceptual variations are unlikely to have confounded the cross-task decoding results, future studies should consider harmonizing stimulus types across tasks to further minimize potential perceptual confounds and confirm the robustness of our findings.

## Resource availability

### Lead contact

Further information and requests for resources should be directed to and will be fulfilled by the lead contact, Bijuan Huang (huang_bijuan0916@163.com).

### Materials availability

This study did not generate new materials.

### Data and code availability

• Data: EEG data are available at https://doc.ml.tu-berlin.de/simultaneous_EEG_NIRS/.
• Code: code for data processing and analysis is publicly available at https://github.com/JaeyoungShin/simultaneous_EEG-NIRS.
• Additional information: any additional information that is required to analyze the data reported in the article can be obtained by contacting the lead contact.

## Acknowledgments

This work was supported by the 10.13039/501100007129Shandong Provincial Natural Science Foundation (ZR2025QC316) and Anhui 10.13039/100016069Philosophy and Social Science Planning Project (AHSKQ2024D056; Recipient: Shuoqi Xiang).

## Author contributions

Conceptualization: W.Y., S.X., and B.J.; methodology: W.Y.; formal analysis: W.Y.; writing – original draft: W.Y.; writing – review and editing: S.X. and B.J.; Supervision: S.X. and B.J.

## Declaration of interests

The authors declare no competing interests.

## STAR★Methods

### Key resources table

**Table:** 

REAGENT or RESOURCE	SOURCE	IDENTIFIER
**Deposited data**		

EEG data of 26 human subjects	Shin et al.^58^	https://doc.ml.tu-berlin.de/simultaneous_EEG_NIRS/

**Software and algorithms**		

MATLAB 2023a	Mathworks	https://www.mathworks.com
Python 3.12	Python Software Foundation	https://www.python.org/
EEGLAB toolbox	Delorme and Makeig^108^	https://doi.org/10.1016/j.jneumeth.2003.10.009
Fieldtrip toolbox	Oostenveld et al.^109^	https://doi.org/10.1155/2011/156869
R (Version 4.5.1)	R Core Team^110^	https://www.R-project.org/
bruceR (Version 4.5.1)	Bao^111^	https://cran.r-project.org/package=bruceR
NeuroRA toolbox	Lu and Ku^112^	https://doi.org/10.3389/fninf.2020.563669

**Other**		

Code	N/A	https://github.com/JaeyoungShin/simultaneous_EEG-NIRS

### Experimental model and study participant details

A total of 26 participants (age = 26.1 ± 3.5 years; 9 males) took part in this study. All were right-handed and reported no neurological, psychiatric, or other brain-related disorders that could affect the results. This study was approved by the Ethics Committee of the Institute of Psychology and Human Factors Engineering, Berlin Institute of Technology (approval number: SH_01_20150330).

### Method details

#### Apparatus and EEG recordings

EEG data were recorded using a multichannel BrainAmp EEG amplifier (Brain Products GmbH, Gilching, Germany) at a sampling rate of 1,000 Hz. Thirty EEG active electrodes were placed on a stretchy fabric cap (EASYCAP GmbH, Herrsching am Ammersee, Germany) according to the international 10-5 system (Fp1, Fp2, AFF5h, AFF6h, AFz, F1, F2, FC1, FC2, FC5, FC6, Cz, C3, C4, T7, T8, CP1, CP2, CP5, CP6, Pz, P3, P4, P7, P8, POz, O1, O2, TP9 (reference) and TP10 (ground)). The continuous EEG data were downsampled offline to 200 Hz. For additional technical details, see Shin et al.^58^

#### Experimental procedure and stimuli

Participants were seated comfortably in an armchair, with their eyes positioned approximately 1.2 m from a 24′ LCD monitor used for stimulus presentation. The display had a refresh rate of 50 Hz, with white stimuli presented on a black background. All stimuli were centrally displayed on the screen to ensure consistent visual presentation across trials.

Figure 1 illustrates the procedures of the n-back and oddball tasks. For the n-back task, participants completed three sessions, each consisting of three series of 0-back, 2-back, and 3-back tasks arranged in a counterbalanced order (i.e., 0→2→3→2→3→0→3→0→2). Each participant thus completed a total of nine series. Each series comprised three segments: a 2s instruction indicating the task type (0-, 2-, or 3-back), a 40s task period, and a 20s rest period. A brief beep (250 ms) signaled the beginning and end of the task period, and the word “STOP” was additionally displayed on the monitor for 1s at the end of the task period. During the rest period, a fixation cross was presented on the monitor. During the task period, a random single-digit number was presented every 2s. Each number was displayed for 0.5s, followed by a fixation cross for the remaining 1.5s. This sequence was repeated for 20 trials, with the target probability set at 30% (70% nontarget). In the 0-back task (control condition), participants responded by pressing the “target” button (number 7) with their right index finger or the “nontarget” button (number 8) with their right middle finger to ensure participant engagement. In the 2- or 3-back tasks, if the current number matched the number presented 2 or 3 trials earlier, respectively, participants responded by pressing the “target” button; if not, they pressed the “nontarget” button. During the rest period, the fixation cross was displayed for 20s, and participants were instructed to relax and maintain fixation to allow the brain activity to return to baseline and to minimize eye movements. A total of 180 trials were conducted for each n-back task (i.e., 20 trials × 3 series × 3 sessions).

For the oddball task, the experiment consisted of three sessions, each containing three task sequences arranged in counterbalanced order. Each participant completed nine series of the task. Each trial consisted of a 2s instruction displaying “O: press button” on the monitor, a 40s task period, and a 20s rest period, with a fixation cross presented in the center of the monitor. The task period was signaled by a brief beep (250 ms) at the beginning and another brief beep (250 ms) at the end, with the word “STOP” displayed for 1s on the screen. During the task period, the symbols “O” and “X” were presented in a random order every 2s. Each symbol was displayed for 0.5s, with a fixation cross displayed for the remaining 1.5s. This sequence was repeated for 20 trials, with “O” appearing on 30% of trials and “X” on 70%. Once the symbol “O” appeared, participants pressed the “target” button (number 7) with their right index finger. If the symbol “X” was displayed, participants pressed the “nontarget” button (number 8) with their right middle finger. This ensured that participants could focus on the task, and response speed was not emphasized. During the rest period, participants performed the same procedure as in the n-back task by relaxing and fixating on the fixation cross. A total of 180 trials were conducted (i.e., 20 trials × 3 series × 3 sessions).

#### EEG data preprocessing

The EEG data were preprocessed using the EEGLAB 2021.1 toolbox^108^ on the MATLAB 2023a platform. The continuous EEG data were high-pass filtered at 1 Hz and low-pass filtered at 30 Hz using the *pop_eegfiltnew* function (default options: zero-phase Hamming-windowed sinc FIR filter). Epochs were defined from −200 ms to 1000 ms relative to stimulus onset. Trials contaminated by eye blinks, muscle, cardiac, and other transient artifacts were corrected using independent component analysis (ICA) via the built-in function *pop*_*runica* of the EEGLAB toolbox. Artifactual components were carefully visually detected based on the time course, topography, and power spectral density of the components. Baseline correction was performed using the period from −200 ms to 0 ms. Electrodes with excessively noisy signals were interpolated using spherical spline interpolation.^113^ Epochs with amplitudes exceeding ±100 μV were discarded. Only trials with correct responses were included in the analysis. For the oddball task, the remaining mean number of trials was 107 (*SD* = 1.72) for oddball and 249 (*SD* = 3.48) for standards. For the n-back task, the remaining mean trial numbers were as follows: 0-back target = 175 (*SD* = 9.88); 2-back nontarget = 99 (*SD* = 11.39); 2-back target = 47 (*SD* = 5.99); 3-back nontarget = 86 (*SD* = 8.92); 3-back target = 39 (*SD* = 7.29). Finally, a common average reference was applied to the data before subsequent analyses.

#### Multivariate pattern analysis

MVPA was conducted using the NeuroRA toolbox,^112^ with a support vector machine (SVM) serving as the classification algorithm. Within-task decoding was first performed as a foundation for subsequent cross-task decoding. Taking the oddball task (oddball vs. standard) as an example, a time-by-time decoding analysis was conducted. To improve decoding efficiency and performance,^114^^,^^115^^,^^116^ the EEG data were downsampled by averaging every two time points, thereby reducing the sampling rate from 200 Hz to 100 Hz. The time-by-time decoding analysis procedure included the following steps: (1) For conditions with unequal numbers of trials, an under-sampling approach was applied. In each iteration, before training the SVM, the condition with fewer trials was first identified. Then, to balance the sample sizes, an equal number of trials were randomly selected from the condition with more trials so that both conditions could be used to train the classifier with matched trial counts. This step was intended to reduce decoding bias caused by the initial sample imbalance. Subsequently, for each condition, the trial order was randomized, and pseudo-trials were constructed by averaging every 4 trials to increase the signal-to-noise ratio and decoding accuracy. The choice of averaging every 4 trials was informed by previous research indicating that modest trial averaging provides an effective trade-off between improving signal-to-noise ratio and maintaining a sufficient number of trials for accurate classification.^51^^,^^57^ (2) A 4-fold cross-validation (CV) was then performed, where 3/4 of the samples were used for training and the remaining 1/4 for testing. This process was repeated four times so that each subset was used as a test set once.^117^^,^^118^ (3) To obtain robust decoding accuracy, steps (1) and (2) were repeated 100 times, and the results were averaged. The choice of 100 repetitions was based on previous studies, which demonstrated that this number offers a good trade-off between computational efficiency and the stability of decoding performance.^52^^,^^56^^,^^116^

Next, the temporal generalization analysis (TGA) was conducted to assess the temporal stability of neural activation patterns.^54^ The TGA procedure followed the same steps as the time-by-time decoding analysis, except that the classifier was tested not only at the training time point but also at all other time points, generating a temporal generalization matrix. The diagonal of this matrix corresponds to the results of the time-by-time decoding analysis, while the off-diagonal elements reflect the temporal evolution of neural activation patterns.

For the n-back task, MVPA was performed for all condition pairs (see Table 1) using the same procedure described above. Notably, the n-back conditions were categorized based on whether there was memory load (i.e., with-load) or not (i.e., load-free). This distinction was based on prior findings showing that P300 amplitude is sensitive to WM load and tends to decrease as WM load increases.^11^^,^^12^^,^^119^

In addition to examining the decoding performance at the global level (i.e., using all electrodes as features) as described above, this study also investigated the decoding effects at the local level, focusing on specific brain networks. Based on the electrode proximity, six networks were defined: frontal, central, parietal/occipital, frontal-central, frontal-parietal/occipital, and central-parietal/occipital. The first three represent within-network configurations, while the latter three represent between-network configurations (see Figure 3C). This division was informed by prior studies showing that P300 elicited by oddball and n-back tasks is typically distributed across frontal and parietal regions, forming an anterior-posterior gradient.^1^^,^^65^^,^^90^ This division enables a more fine-grained characterization of the brain networks underlying the P300, thereby enhancing the robustness of the conclusions in this study.

Importantly, to examine whether the oddball and n-back tasks share common neural mechanisms, cross-task decoding analysis was performed. The procedure was largely similar to that used in TGA, except that the training set and testing set were derived from two different tasks. Specifically, classifiers were trained at each time point on data from the oddball task and tested across all time points on data from the n-back task, yielding a temporal generalization matrix (matrix A). This procedure was then reversed: classifiers were trained on data from the n-back task and then tested on data from the oddball task, producing another matrix (matrix B). Matrix A and the transpose of matrix B were then averaged to yield a final measure of the shared neural representations between the two tasks. Moreover, similar to within-task decoding, cross-task decoding analysis was performed at both the global and local levels.

In addition, this study also tested decoding performance using averages of 2 and 6 trials to construct pseudo-trials (see Figures S1–S6). These analyses produced decoding results that were comparable to those obtained with 4-trial averaging, further confirming the robustness of our results.

### Quantification and statistical analysis

For the time-by-time decoding analysis, a cluster-based permutation test (CBPT) was employed to assess statistical significance while correcting for multiple comparisons.^116^^,^^120^ This approach controls for multiple comparisons by identifying clusters of temporally adjacent samples that exceed a predefined statistical threshold (*p* < 0.05), and by evaluating their cluster-level statistics against a null distribution derived from a permutation test. Thus, it inherently accounts for multiple tests across time points. The procedure was as follows: (1) A one-sample *t* test (α = 0.05) was conducted at each time point to compare decoding accuracy against the chance level of 0.5 for binary classification and to identify the significant clusters; (2) For each cluster, the sum of the *t*-values was computed and used as the cluster-level statistic; (3) A null distribution of cluster-level statistics was constructed using the Monte Carlo method (5,000 permutations); (4) By comparing the cluster statistics to the null distribution, the *p*-values (0.05, one-sided) were obtained. The same procedure was applied to test the significance of the TGA results. This approach helps us determine the statistical significance of the observed decoding accuracy while accounting for multiple comparisons.

Furthermore, for the significant decoding results among the six brain networks, Bonferroni correction was employed to control for multiple comparisons (*p* = 0.05/6 = 0.0083) in step (4). The analysis was performed in NeuroRA.^112^ To further demonstrate that the shared neural mechanism was mainly the P300 rather than the P3a (i.e., the decoding performance of the parietal/occipital network outperformed that of the frontal network), a one-way repeated-measures ANOVA (rmANOVA) was conducted to compare the peak accuracy and its corresponding latency (ms) of cross-task decoding across the frontal, parietal/occipital, and frontal-parietal/occipital networks. The peak accuracies and their corresponding latencies were extracted from the largest significant cluster within the P300 time window. When a significant network effect was observed, post hoc analysis was performed. In the case of violations of sphericity assumptions, a Greenhouse-Geisser correction was applied to the degrees of freedom. To control multiple comparisons, all post hoc tests were Bonferroni corrected. The analysis was performed in bruceR.^111^

For ERP univariate analysis, two complementary statistical approaches were conducted. First, the aforementioned CBPT was performed to assess the significance of P300^120^ with the following modifications: in step (1), a paired-sample *t* test was used to identify the significant clusters. A cluster was formed if at least two adjacent electrodes reached the significance threshold (α = 0.05, two-sided). The adjacency of electrodes was determined using the “triangulation” method. In step (4), a two-tailed test was applied. The analysis was performed in Fieldtrip.^109^ Second, the P300 mean amplitudes at the Pz electrode were extracted and analyzed using a paired-sample *t* test. Bonferroni correction was performed to control multiple comparisons. The analysis was performed in bruceR.^111^

### Additional resources

This study conducted a reanalysis of publicly available data. A full description of the participants, stimuli, tasks, and preprocessing procedures can be found in the original publication.^58^ Here, we provide a brief summary along with a detailed account of the analyses conducted in the present study.
