# Supplementary material for: Decoding P300 as a shared neural mechanism for oddball target detection and working memory updating
Source: iScience. 2026 Jan 21;29(2):114757. doi: 10.1016/j.isci.2026.114757 (PMC12907112; doi:10.1016/j.isci.2026.114757)
Supplement: Document S1. Figures S1–S6 [file mmc1.pdf]

iScience, Volume *29*

Supplemental information

Decoding P300 as a shared neural mechanism for oddball target detection and working memory updating

Weixing Yang, Shuoqi Xiang, and Bijuan Huang

Supplemental information

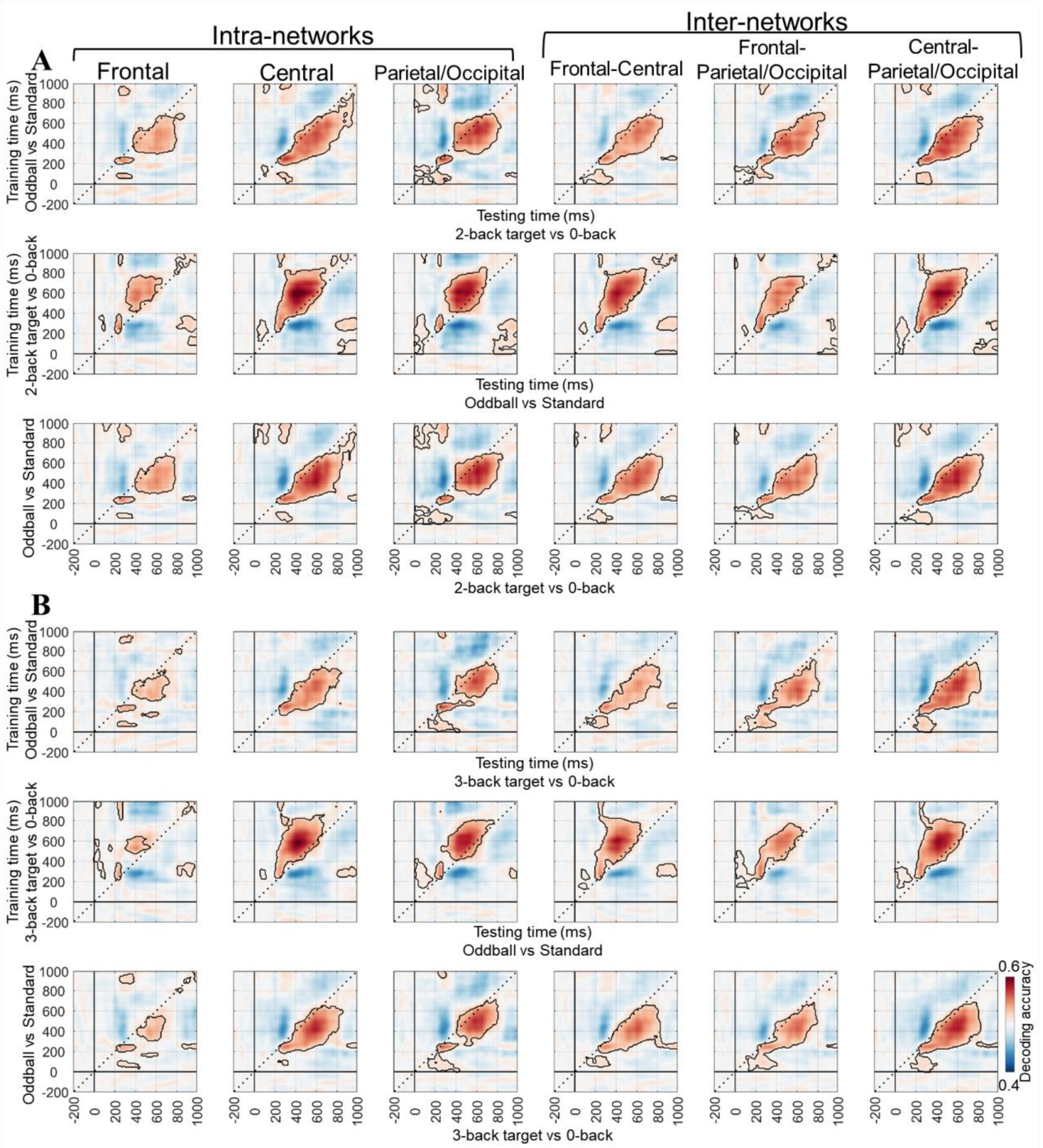

Figure S1. Averaging 2 trials cross-task decoding results under the with-load condition (2-/3-back target vs. 0-back) at the local level (cluster-based permutation test, $p < 0.05/6 = 0.0083$).

(A) The first row shows the results that the oddball task (oddball vs. standard) was used as the training set and the n-back task (2-back target vs. 0-back) as the testing set. The second row presents the reversed direction, with the n-back task used as the training set and the oddball task as the testing set. The third row displays the average of the two matrices above, representing the shared neural representations. Each column represents a specific brain network.

(B) Other explanations are the same as in (A), except for the use of the 3-back task.

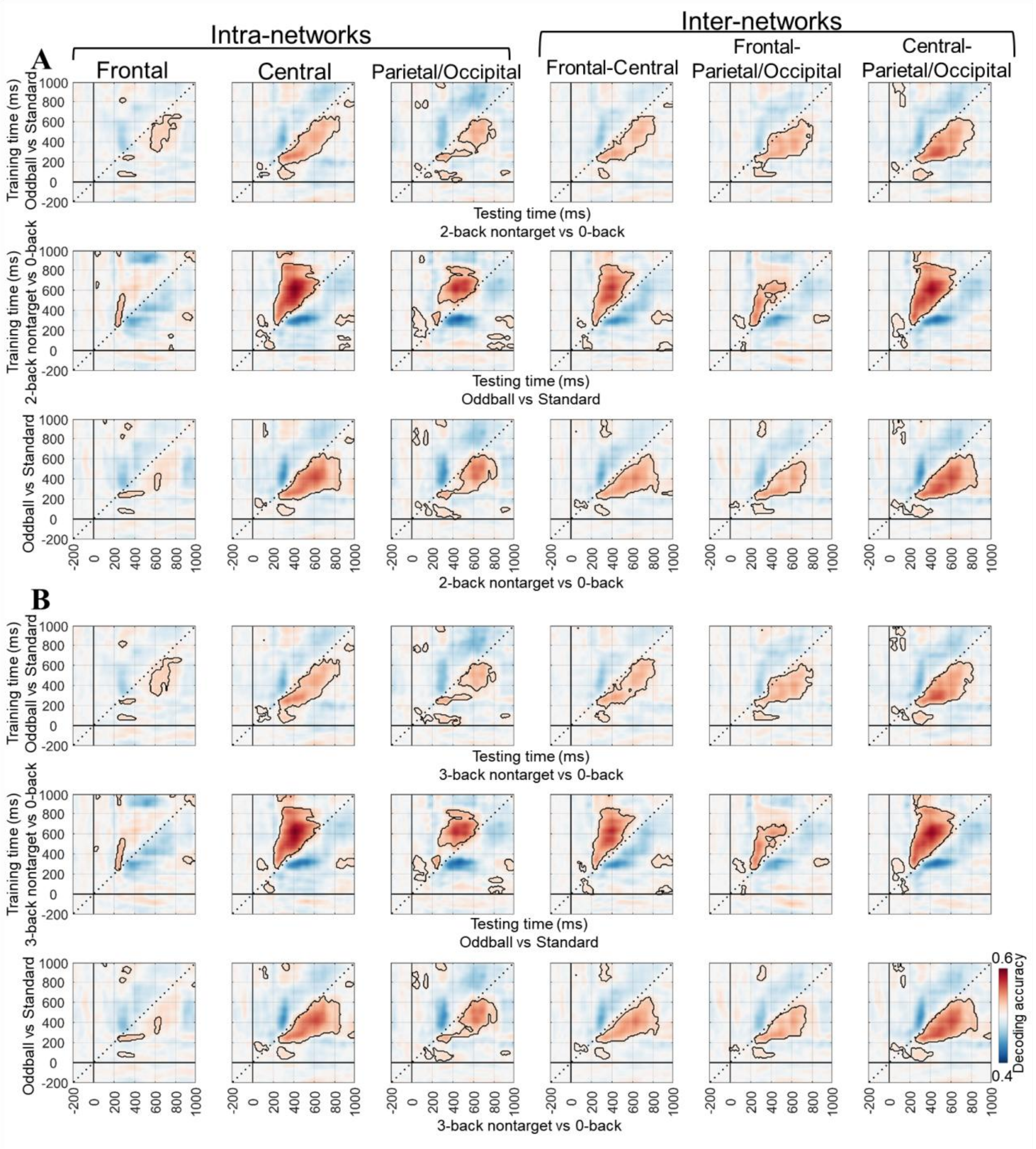

Figure S2. Averaging 2 trials cross-task decoding results under the with-load condition (2-/3-back nontarget vs. 0-back) at the local level (cluster-based permutation test, $p < 0.05/6 = 0.0083$).

(A) The first row shows the results that the oddball task (oddball vs. standard) was used as the training set and the n-back task (2-back target vs. 0-back) as the testing set. The second row presents the reversed direction, with the n-back task used as the training set and the oddball task as the testing set. The third row displays the average of the two matrices above, representing the shared neural representations. Each column represents a specific brain network.

(B) Other explanations are the same as in (A), except for the use of the 3-back task.

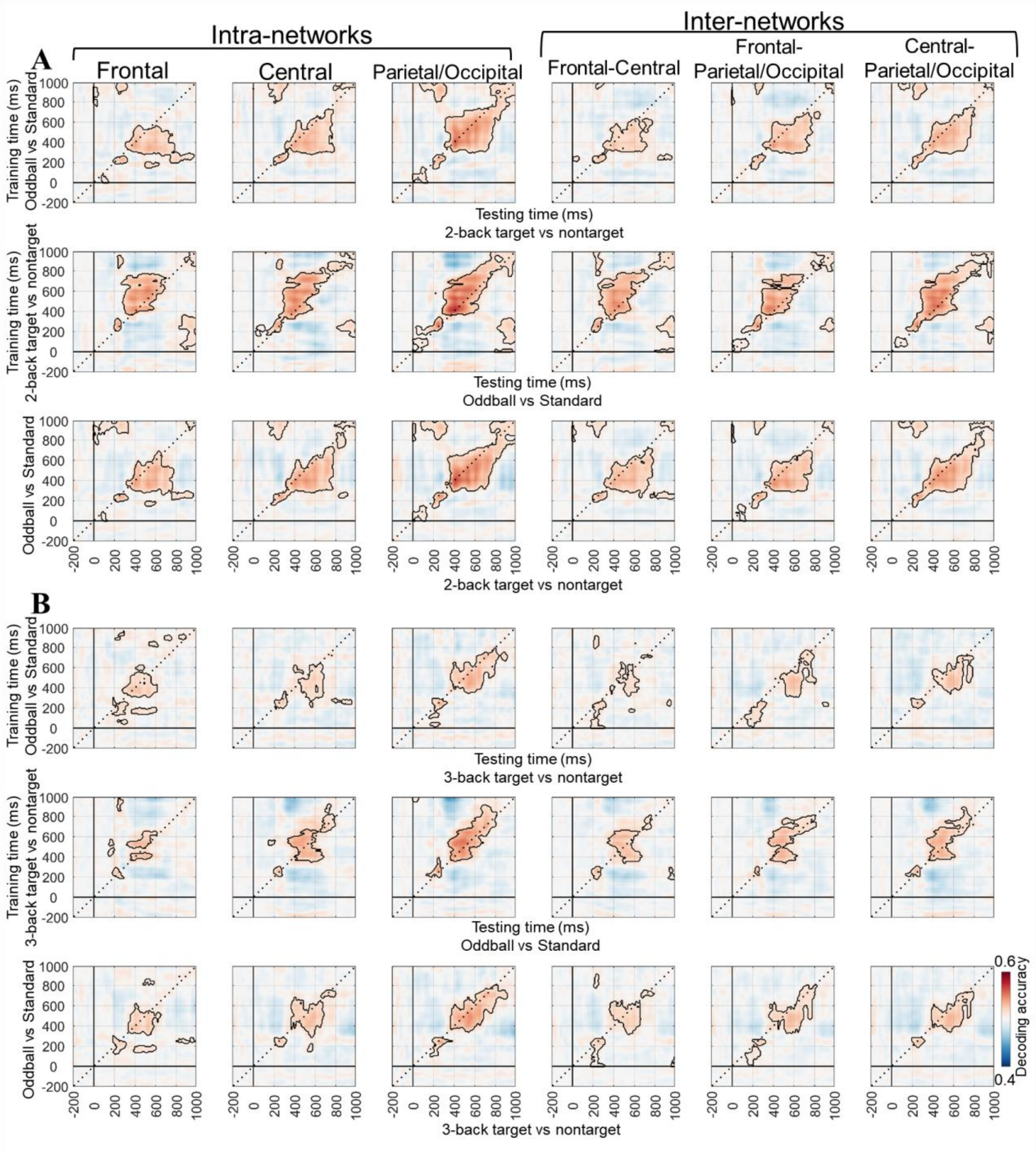

Figure S3. Averaging 2 trials cross-task decoding results under the with-load condition (2-/3-back target vs. nontarget) at the local level (cluster-based permutation test, $p < 0.05/6 = 0.0083$).

(A) The first row shows the results that the oddball task (oddball vs. standard) was used as the training set and the n-back task (2-back target vs. 0-back) as the testing set. The second row presents the reversed direction, with the n-back task used as the training set and the oddball task as the testing set. The third row displays the average of the two matrices above, representing the shared neural representations. Each column represents a specific brain network.

(B) Other explanations are the same as in (A), except for the use of the 3-back task.

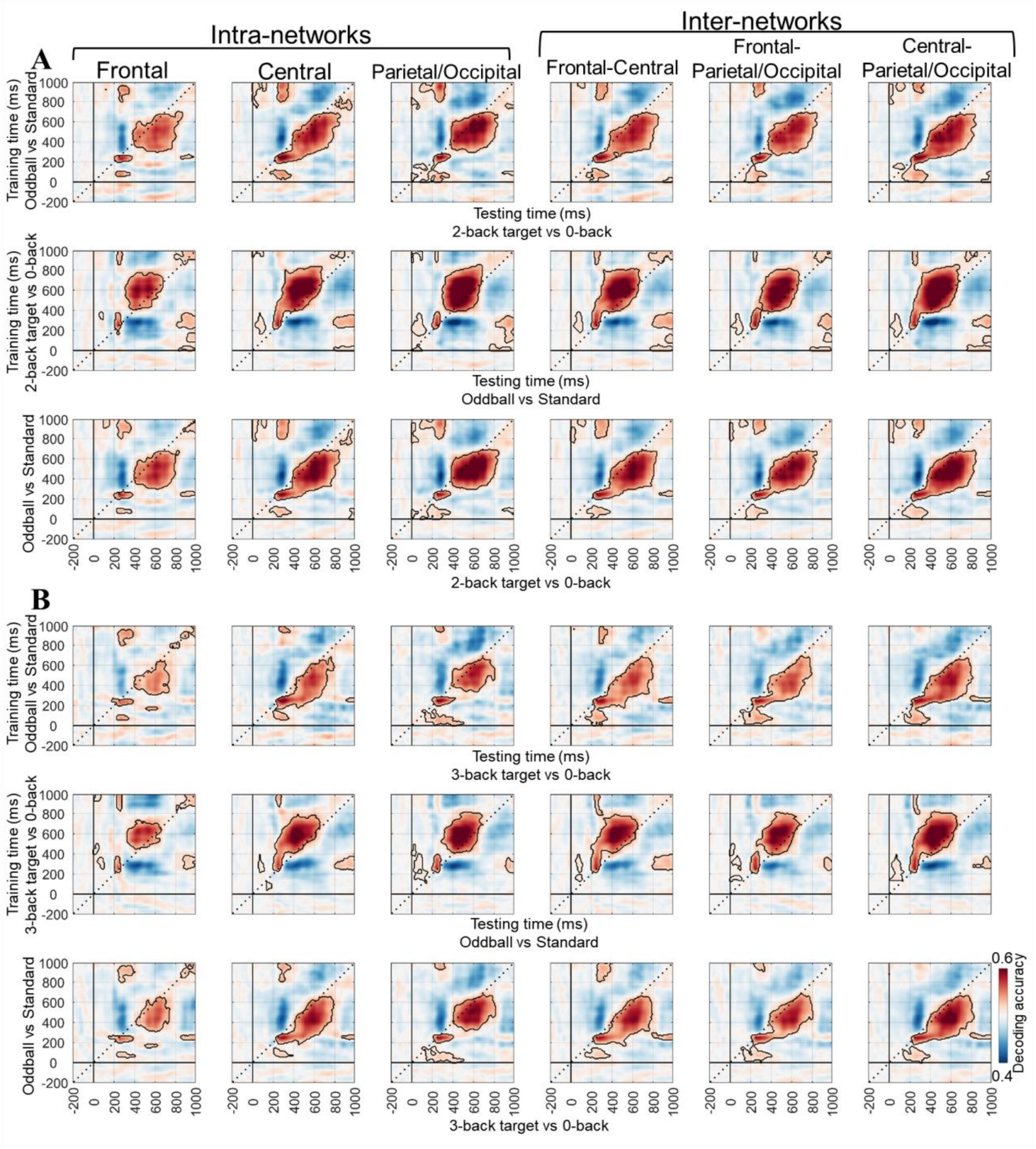

Figure S4. Averaging 6 trials cross-task decoding results under the with-load condition (2-/3-back target vs. 0-back) at the local level (cluster-based permutation test, *p* < 0.05/6 = 0.0083).

(A) The first row shows the results that the oddball task (oddball vs. standard) was used as the training set and the n-back task (2-back target vs. 0-back) as the testing set. The second row presents the reversed direction, with the n-back task used as the training set and the oddball task as the testing set. The third row displays the average of the two matrices above, representing the shared neural representations. Each column represents a specific brain network.

(B) Other explanations are the same as in (A), except for the use of the 3-back task.

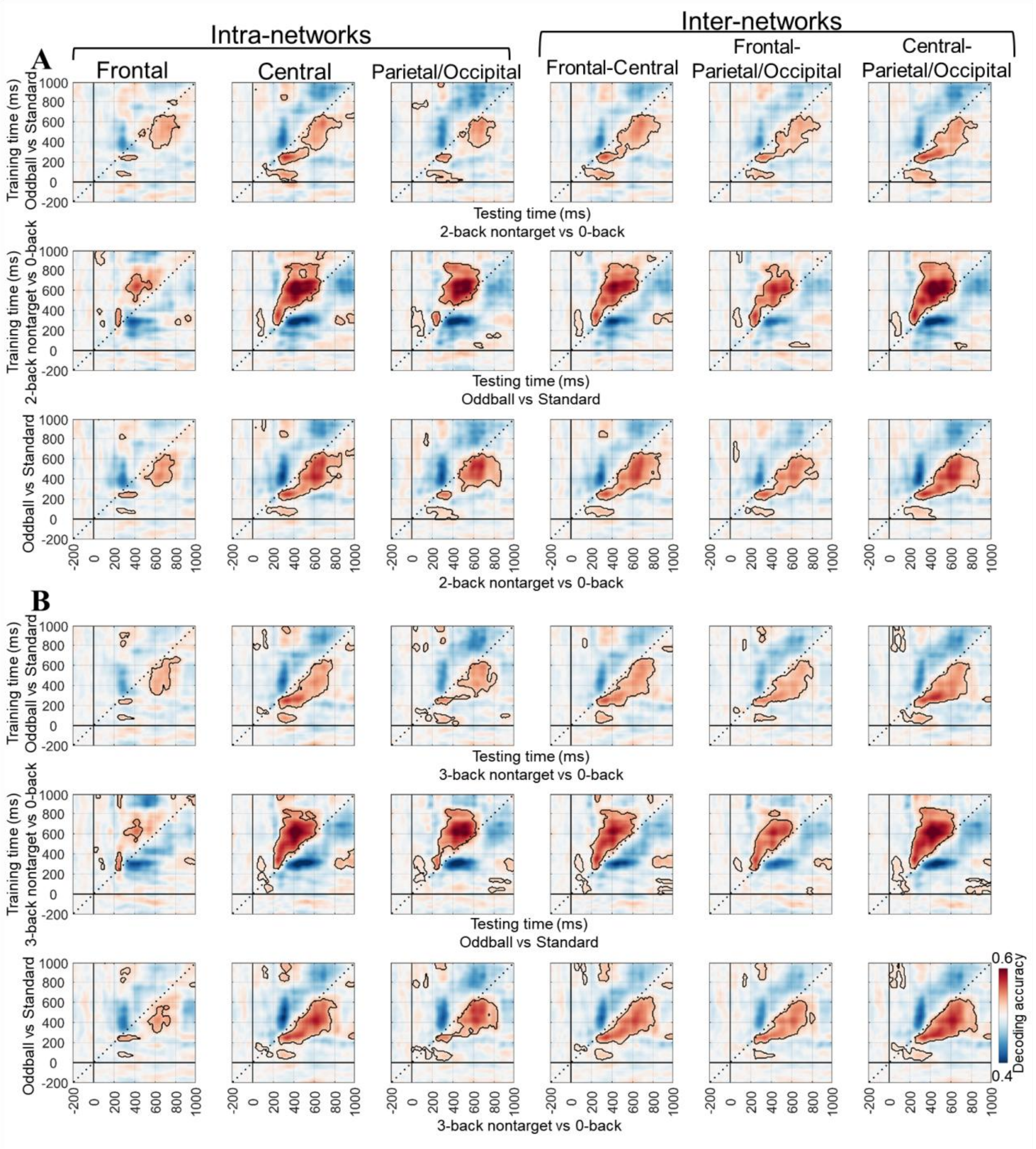

Figure S5. Averaging 6 trials cross-task decoding results under the with-load condition (2-/3-back nontarget vs. 0-back) at the local level (cluster-based permutation test, $p < 0.05/6 = 0.0083$).

(A) The first row shows the results that the oddball task (oddball vs. standard) was used as the training set and the n-back task (2-back target vs. 0-back) as the testing set. The second row presents the reversed direction, with the n-back task used as the training set and the oddball task as the testing set. The third row displays the average of the two matrices above, representing the shared neural representations. Each column represents a specific brain network.

(B) Other explanations are the same as in (A), except for the use of the 3-back task.

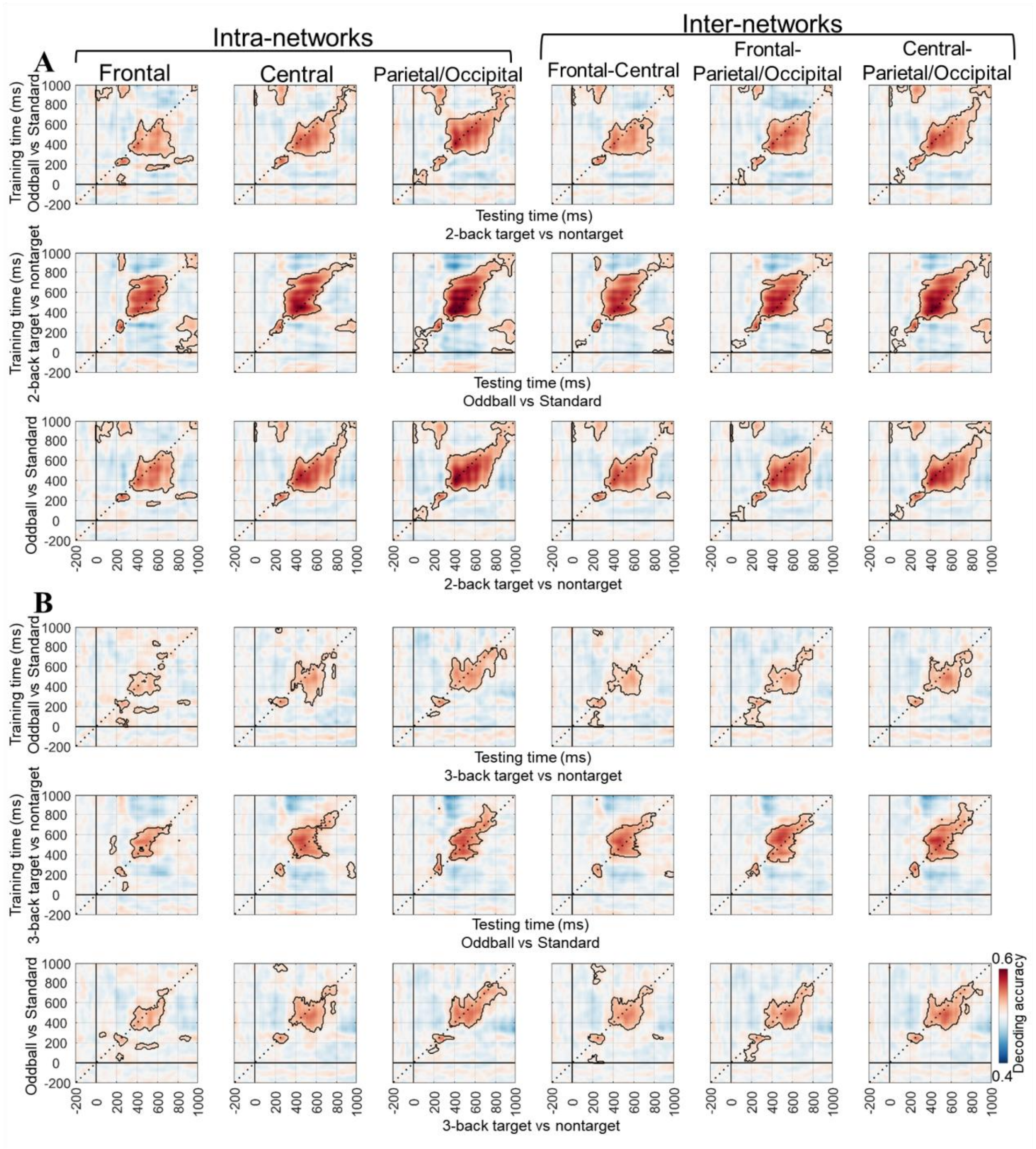

Figure S6. Averaging 6 trials cross-task decoding results under the with-load condition (2-/3-back target vs. nontarget) at the local level (cluster-based permutation test, $p < 0.05/6 = 0.0083$).

(A) The first row shows the results that the oddball task (oddball vs. standard) was used as the training set and the n-back task (2-back target vs. 0-back) as the testing set. The second row presents the reversed direction, with the n-back task used as the training set and the oddball task as the testing set. The third row displays the average of the two matrices above, representing the shared neural representations. Each column represents a specific brain network.

(B) Other explanations are the same as in (A), except for the use of the 3-back task.
